# Immune evasion by proteolytic shedding of natural killer group 2, member D ligands in *Helicobacter pylori* infection

**DOI:** 10.3389/fimmu.2024.1282680

**Published:** 2024-01-22

**Authors:** Margit Anthofer, Markus Windisch, Rosa Haller, Sandra Ehmann, Sebastian Wrighton, Michael Miller, Lorenz Schernthanner, Iris Kufferath, Silvia Schauer, Barbara Jelušić, Sabine Kienesberger, Ellen L. Zechner, Gernot Posselt, Mar Vales-Gomez, Hugh T. Reyburn, Gregor Gorkiewicz

**Affiliations:** ^1^Institute of Pathology, Medical University of Graz, Graz, Austria; ^2^Institute of Molecular Biosciences, University of Graz, Graz, Austria; ^3^Interuniversity Cooperation, BioTechMed-Graz, Graz, Austria; ^4^Department of Biosciences and Medical Biology, Paris Lodron University of Salzburg, Salzburg, Austria; ^5^Department of Immunology and Oncology, Spanish National Centre for Biotechnology, Madrid, Spain

**Keywords:** *H. pylori*, immune evasion, NK cells, cytotoxic T cells, stomach cancer, tumor immunity, stomach microbiota, NKG2D (natural killer group 2 member D)

## Abstract

**Background:**

*Helicobacter pylori* (*H. pylori*) uses various strategies that attenuate mucosal immunity to ensure its persistence in the stomach. We recently found evidence that *H. pylori* might modulate the natural killer group 2, member 2 (NKG2D) system. The NKG2D receptor and its ligands are a major activation system of natural killer and cytotoxic T cells, which are important for mucosal immunity and tumor immunosurveillance. The NKG2D system allows recognition and elimination of infected and transformed cells, however viruses and cancers often subvert its activation. Here we aimed to identify a potential evasion of the NKG2D system in *H. pylori* infection.

**Methods:**

We analyzed expression of NKG2D system genes in gastric tissues of *H. pylori* gastritis and gastric cancer patients, and performed cell-culture based infection experiments using *H. pylori* isogenic mutants and epithelial and NK cell lines.

**Results:**

In biopsies of *H. pylori* gastritis patients, NKG2D receptor expression was reduced while NKG2D ligands accumulated in the lamina propria, suggesting NKG2D evasion. *In vitro*, *H. pylori* induced the transcription and proteolytic shedding of NKG2D ligands in stomach epithelial cells, and these effects were associated with specific *H. pylori* virulence factors. The *H. pylori*-driven release of soluble NKG2D ligands reduced the immunogenic visibility of infected cells and attenuated the cytotoxic activity of effector immune cells, specifically the anti-tumor activity of NK cells.

**Conclusion:**

*H. pylori* manipulates the NKG2D system. This so far unrecognized strategy of immune evasion by *H. pylori* could potentially facilitate chronic bacterial persistence and might also promote stomach cancer development by allowing transformed cells to escape immune recognition and grow unimpeded to overt malignancy.

## 1 Introduction

*Helicobacter pylori* (*H. pylori*) is a major human pathogen causing chronic gastritis and gastroduodenal ulcer disease. It also drives the development of gastric adenocarcinoma and MALT lymphoma and is considered a class-I carcinogen (1). *H. pylori* infection typically occurs early in life (2), and persists life-long unless eradicated therapeutically (1). To establish chronic infection and mucosal persistence, *H. pylori* has evolved multiple strategies to overcome immunity (3). These strategies include avoiding recognition by TLRs, surviving phagocytosis by neutrophils and macrophages, modifying expression of immunomodulatory proteins (e.g. PD-L1) in stomach epithelial cells and modulating lymphocyte activation, proliferation and differentiation (1, 3, 4). Noteworthy, the induction of certain anti-inflammatory circuits in *H. pylori* infection, such as the promotion of interleukin 10 (IL-10)-producing regulatory T cells (Tregs), benefits the host by counteracting the development of atopic diseases like asthma (3, 5). However, weakened immunity also favors cancer development since transformed cells may escape immune surveillance and produce overt cancer.

The natural killer group 2, member D (NKG2D) system is a well characterized immune surveillance system, which is important for the elimination of infected and transformed cells (6). The NKG2D receptor is expressed on natural killer (NK) cells, cytotoxic T lymphocytes (CTLs) and gamma delta T cells (γδ T cells) (7, 8), all of which are part of mucosal immunity (9, 10). NKG2D recognizes the NKG2D ligands (NKG2D-Ls) MHC class I polypeptide-related sequence A (MICA), MHC class I polypeptide-related sequence B (MICB) and UL16 binding proteins 1-6 (ULBP 1-6) (6), which are mainly expressed intracellularly by epithelia and certain other cell types (11, 12). Cell surface expression of NKG2D-Ls is specifically increased under stress conditions such as infection (8, 13, 14), or oncogenic transformation (15). The receptor-ligand interaction, then, activates the cytotoxic immune cells to eliminate the ligand-expressing cells (7, 8, 13, 16). Notably, some viruses but also many cancers escape this mechanism, either by downregulating NKG2D-L expression or by releasing NKG2D-Ls as soluble proteins from the cell surface, which reduces the cells’ immunological visibility (17–19). Soluble NKG2D-Ls additionally attenuate the strength of the immune response by acting as suppressors of NKG2D-expressing effector cells. Their binding to the receptor leads to reduced NKG2D expression and thus diminished NKG2D-mediated immunity and an overall hypofunctional immune cell phenotype (20–23).

Several studies indicate an important role for the NKG2D system in mucosal homeostasis and the microbiota and certain microbial metabolites (e.g. short-chain fatty acids; SCFAs) are able to modulate NKG2D-L expression in the gut (14, 24–26). Interestingly, the system is dysregulated and contributes to inflammation in diseases such as celiac disease (27), and Crohn’s disease (28), conditions where *H. pylori* infection seems to be beneficial (5). We recently found indications that *H. pylori* might modulate the NKG2D system (26). Here we aimed to investigate this finding in more detail. By analyzing human stomach biopsies from *H. pylori* gastritis and gastric cancer cases and by employing cell-culture based infection models with epithelial and NK cells we demonstrate that *H. pylori* can actively subvert the NKG2D system. This so far unrecognized immune evasion strategy could potentially help the pathogen to persist in the stomach mucosa, and might also favor stomach cancer development.

## 2 Materials and methods

### 2.1 Human stomach biopsies

Formalin-fixed and paraffin-embedded (FFPE) tissue samples were derived from the archives of the Institute of Pathology at the Medical University of Graz (**Supplementary Table 1**). *H. pylori* presence was determined by Warthin–Starry staining (29), and IHC with an anti-*H. pylori* antibody (clone SP48; Ventana). Tissue use was approved by the institutional review board of the Medical University of Graz (EK-23-212ex10/11).

### 2.2 Histopathology and IHC

FFPE tissues were subjected to standard H&E staining. For immunohistochemistry, 2 μm thick sections were stained with antibodies against CD45 (Clones 2B11 + PD7/26, Cat# GA75161-2, Agilent Technologies, RRID: AB_2661839), CD8 (Clone C8/144B, Cat# GA62361-2, Agilent Technologies), CD56 (clone MRQ42; 760-4596, Ventana Medical Systems) and NKp46 (clone 195314, MAB1850, R and D systems, RRID: AB_2149153). For CD45 and CD8 detection, slides were stained on a Dako Omins machine using the EnVision FLEX staining system with Antigen-Retrieval using EnVision FLEX TRS, High pH for 30 min at 97°C for CD45 and using EnVision FLEX TRS, Low pH for 24 min at 95°C for CD8 detection. Blocking was achieved with the EnVision FLEX Peroxidase-Blocking-Reagent for 3 min, primary antibodies were incubated for 20 min at 36°C and for detection, the EnVision FLEX HRP and the EnVision FLEX Mouse Linker were used. For detection of CD56 and NKp46, slides were stained with the Ventana system using CC1 Standard (Ventana) at 95°C for 32 min for CD56 and at 95°C for 64 min for NKp46 detection. The CD56 antibody was ready-to-use and was incubated at 36°C for 16 min and the NKp46 antibody was diluted 1:100 and incubated at 36°C for 1 h. For detection, the ultraView Universal DAB Detection Kit (Ventana) was used. For MICA/B detection, antigen retrieval was performed in a microwave in sodium citrate buffer, pH 6.0, (Gatt-Koller) for 40 min. Endogenous peroxidase activity was inhibited with 3% H_2_O_2_ in methanol for 15 min. For blocking of nonspecific protein binding and for detection the UltraVision LP Detection System HRP Polymer (Ready-to-use) (TL-060-HL; Thermo Scientific) was used. Sections were incubated with primary MICA/B antibody (clone F-6; dilution 1:100; Santa Cruz Biotech, RRID: AB_2143751) for 1 h at RT. The sections were counterstained with Mayer’s hemalum solution (Merck) and Entellan (Merck) was used as mounting medium. A pathologist (GG) evaluated and scored all immunohistochemistry. CD45, CD8, CD56 and NKP46 were evaluated by quantifying positive cells per 1 mm^2^ of the tissue section. MICA/B staining was scored by assessing the intensity of staining in epithelial cells and in the lamina propria (0= absent, 0.5= very weak, 1= weak, 1.5= weak to medium, 2= medium, 2.5= medium to strong, 3= strong). In addition, individual snap-frozen (2-methylbutane) stomach biopsy sections were subjected to MICA/B staining.

### 2.3 qPCR

**Figure 1:**
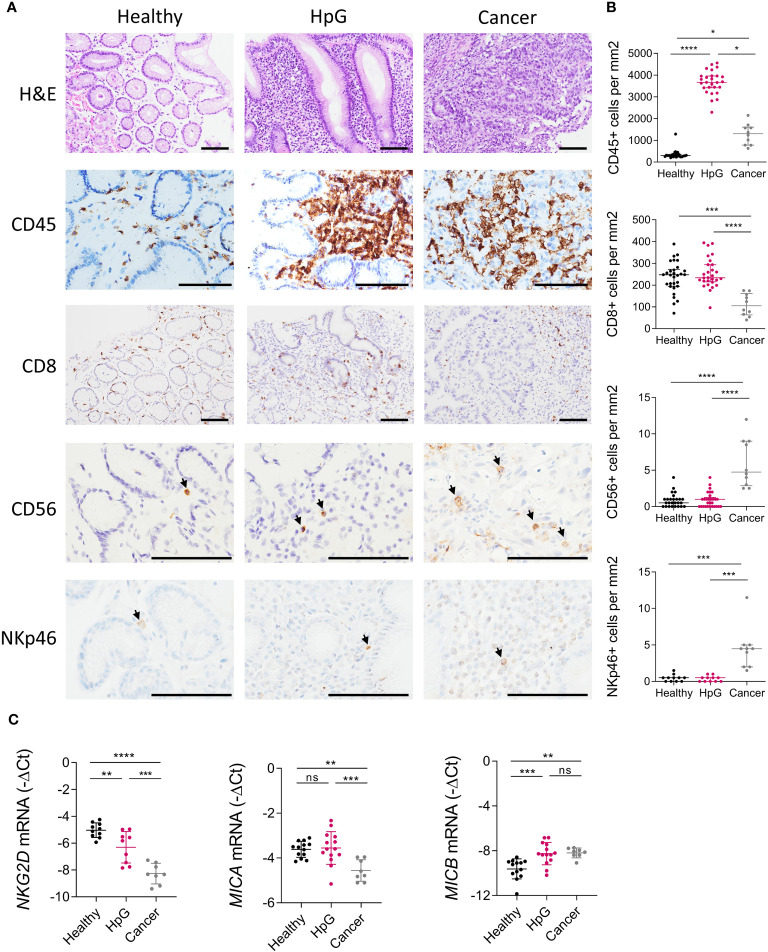
Inflammation and the NKG2D system in *H. pylori* infection and gastric cancer. Stomach biopsies from healthy controls (Healthy), *H. pylori* gastritis cases (HpG) and stomach adenocarcinoma cases (Cancer) were immune-phenotyped. **(A, B)** H&E and IHC staining of leucocytes (CD45), CTLs (CD8) and NK cells (CD56, NKp46). **(A)** Representative images, scale bars: 100 µm. **(B)** Quantification of positive cells per mm^2^ of tissue n=10-30 per group, the data do not follow a normal distribution, median ± interquartile range, Kruskal-Wallis test and Dunn’s test. **(C)** qPCR analysis of *NKG2D*, *MICA* and *MICB*, n=8-16 per group. The data passed Shapiro-Wilk normality test, mean ± SD, one-way ANOVA and Tukey’s test. *P <0.05; **P <0.01; ***P <0.001; ****P <0.0001. ns, not significant.

Total RNA from FFPE samples (7 sections, each 5 µm thick) was isolated with Deparaffinization Solution (Qiagen) and the RNeasy FFPE kit, which includes a DNase treatment step (Qiagen). Total RNA from cell cultures was isolated using the NucleoSpin^®^ RNA extraction kit (Machery-Nagel) or TRIzol reagent (Invitrogen). RNA quality and quantity were determined spectrophotometrically with the NanoDrop™ 2000c (Thermo Fisher Scientific). For cDNA synthesis, the High Capacity cDNA Reverse Transcription kit (Applied Biosystems) and the RNAase inhibitor (Applied Biosystems) were used. qPCR was performed with a CFX384 qPCR thermocycler (BioRad) using SYBR^®^ Green PCR Master Mix (Applied Biosystems). A standard protocol for SYBR^®^ Green was used with 10 min at 95°C, 39 cycles of 15 sec at 97°C and 1 min at 60°C, followed by 15 sec at 60°C and a final melting step until 95°C. The oligonucleotide primers used in this study are listed in **Supplementary Table 2**. Glyceraldehyde 3-phosphate dehydrogenase (GAPDH) was used as reference gene. qPCR data are reported as –ΔCt in **Figure 1C** and as relative expression ratio in all other figures. The relative expression ratio was calculated according to Pfaffl’s method (equation 1) (30). As control group for the calculation of the relative expression ratio we used cells harvested at time point 0 h.

### 2.4 Cell lines

The human stomach epithelial cell line AGS (female, ATCC Number: CRL-1739, RRID: CVCL_0139) was obtained from Cell Lines Service (Eppelheim, Germany) and the human stomach epithelial cell line MKN28 (female, RRID : CVCL_1416) was kindly provided by Dr. Silja Wessler (Department of Biosciences and Medical Biology, Paris Lodron University of Salzburg, Austria) and originally obtained from the Japanese Collection of Research Bioresources (JCRB; http://cellbank.nibio.go.jp/). Adhesion of *H. pylori* to these cell lines was confirmed and cellular responses to the infection were characterized by Schneider et al. (31). AGS and MKN28 cells were cultivated in RPMI 1640 medium (Gibco), supplemented with 2 mmol/L L-glutamine (Gibco), 5 mmol/L HEPES (Gibco) and 10% fetal bovine serum (FBS) (Gibco). The human natural killer cell line NKL (male, RRID: CVCL_0466) was a kind gift of Dr. Francisco Borrego (Biocruces Bizkaia Health Research Institute, Barakaldo, Spain). The chronic myeloid leukemia cell line K562 (female, RRID: CVCL_0004) was obtained from ATCC (cat # CCL-243). NKL and K562 cells were cultured in RPMI 1640 medium, supplemented with 100 units/ml penicillin, 100 mg/ml streptomycin (Gibco), 1 mmol/L sodium pyruvate (Gibco), 0,1 mmol/L nonessential amino acids (Gibco) and 58 µmol/L 2-mercaptoethanol (Sigma Aldrich). K562 cells were additionally supplemented with 10% FBS (Gibco). NKL cells were additionally supplemented with 50 units/ml recombinant human IL-2 (Peprotech), 5% human normal serum (MP Biomedicals™) and 5% FBS (Gibco). All cells were incubated in a water-saturated atmosphere with 5% CO2 at 37°C.

### 2.5 Bacteria

*H. pylori* P12 wild type (expressing Western *cagA* EPIYA-ABCC, and with a *vacA* s1/m1 genotype) (32, 33), and isogenic mutant strains P12 *ΔcagA*, *ΔcagL* and *ΔvacA* were kindly provided by Dr. Silja Wessler (Department of Biosciences and Medical Biology, Paris Lodron University of Salzburg, Austria). *H. pylori* strains were cultured on agar plates containing 10% horse serum under microaerophilic conditions at 37°C. Source and cultivation of the *C. acnes* PA-2.2 strain were described recently (26).

### 2.6 Infection assay, butyrate treatment and metalloprotease inhibition

AGS, AGS-MICA and MKN28 cells were seeded in 6-well plates and grown for 48 h to 70% confluence. *H. pylori* strains were grown on agar plates for 48 h, then harvested in cell culture medium and used for infection. *C. acnes* was cultured on agar plates for 72 h, then harvested in cell culture medium to an optical density (OD_600_) of 0.1 and further grown for 24 h. Subsequently, cells were infected at multiplicities of infection (MOI) of 50 and incubated for 24 h and 48 h, according to other studies that performed infection experiments with *H. pylori* P12 and these stomach epithelial cell lines (34, 35). These infection conditions induce significant phenotypic changes in epithelia cell lines without causing excessive cell death (**Supplementary Figure 1**). Infection with the *H. pylori* WT strain induced the hummingbird phenotype [induced by CagA (36)] and vacuole formation [induced by VacA (37)] (**Supplementary Figure 12**). These phenotypic changes did not occur when cells were infected with the respective mutant strains (*ΔcagA* and *ΔvacA*). Infection with the *ΔcagL* strain did also *not* induce the hummingbird phenotype, consistent with the notion that CagL is essential for CagA transfer to host cells and the subsequent CagA-dependent cell modifications (38). As control treatment served 2 mmol/L butyrate (Sigma Aldrich), that was shown to induce *MICA/B* expression (26, 39). For metalloprotease inhibition, batimastat (BB94) (Sigma Aldrich) a broad-spectrum inhibitor of MMPs and of ADAM proteases was diluted in DMSO and added to AGS and MKN28 cells at 10 µmol/L (40), at the same time as *H. pylori* or butyrate. As solvent control, only DMSO was added to the cultures.

### 2.7 ELISA

**Figure 2:**
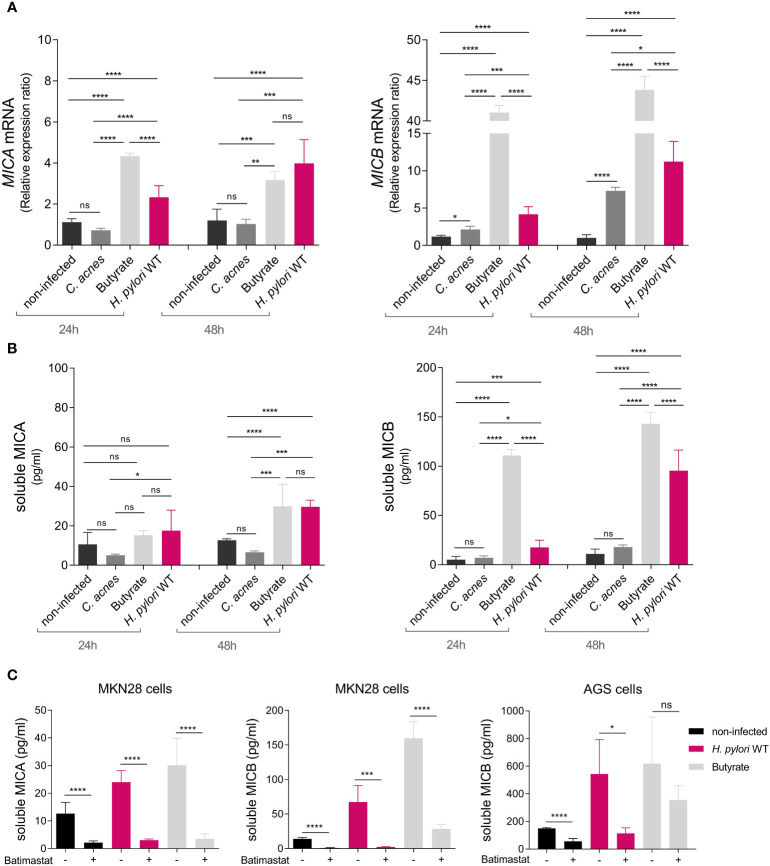
*H. pylori*-dependent modulation of NKG2D-L expression and soluble release via proteolytic shedding. MKN28 cells were challenged with *C. acnes*, butyrate, and *H. pylori* WT for 24 and 48 h **(A)** *MICA* and *MICB* mRNA levels were determined by qPCR. **(B)** Soluble MICA and MICB levels in cell culture supernatants were determined by ELISA. Experiments were performed three times. Mean ± SD, one-way ANOVA and Tukey’s test (ns, not significant, *P <0.05; **P <0.01; ***P <0.001; ****P <0.0001). **(C)** MKN28 and AGS cells were challenged with *H. pylori* WT or 2 mM butyrate for 48 h Simultaneously, cells were treated with either 10 µmol/L batimastat dissolved in DMSO (= batimastat +) or with DMSO alone as solvent control (= batimastat –). Soluble MICA and MICB proteins in cell culture supernatants were determined by ELISA. Experiments were performed three times. Mean ± SD, t-test of batimastat – vs. batimastat +, for each treatment group (ns, not significant, *P <0.05; ***P <0.001; ****P <0.0001).

**Figure 3:**
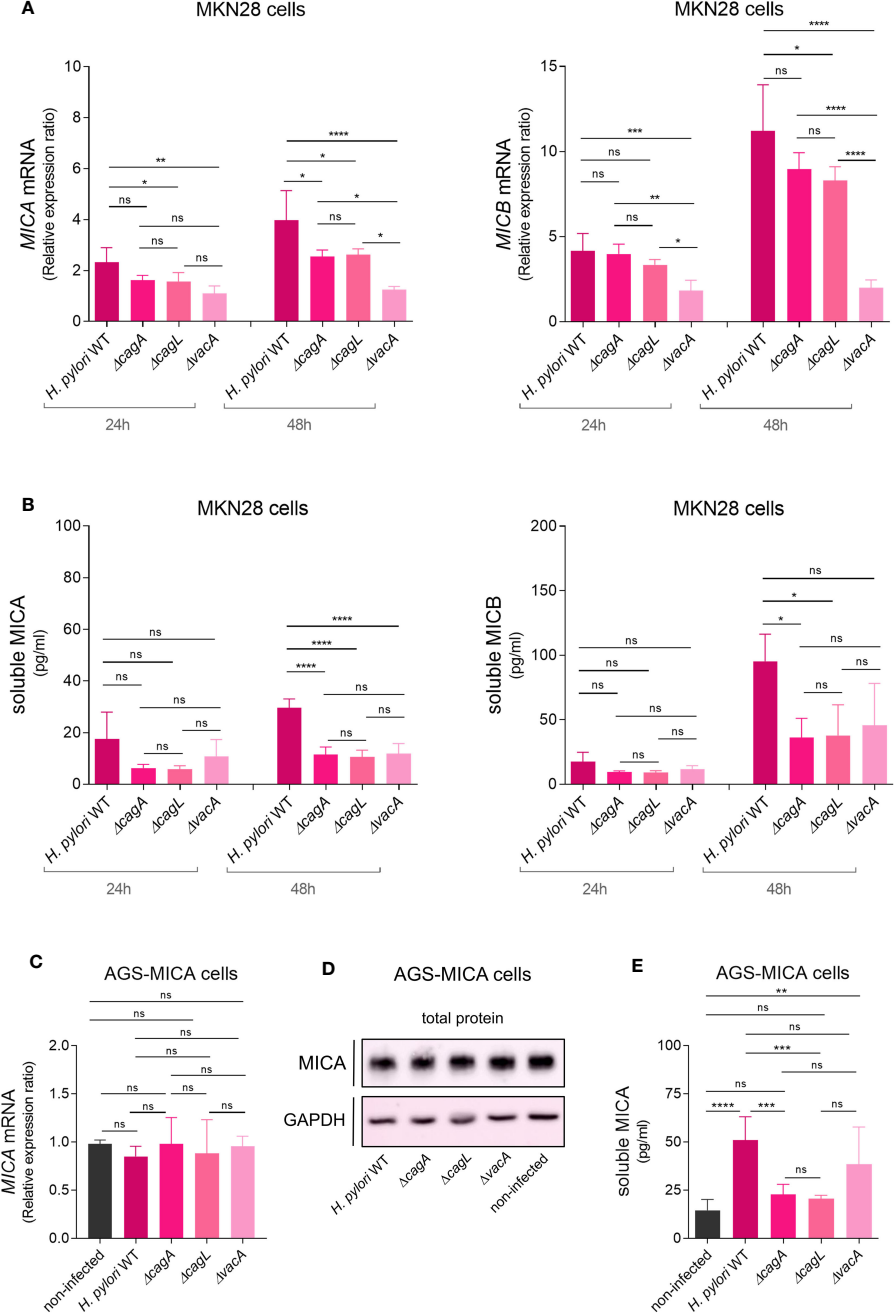
*H. pylori* virulence factor-dependent modulation of NKG2D-L expression and soluble release. MKN28 cells were challenged with *H. pylori* WT and isogenic mutants *ΔcagA*, *ΔcagL* and *ΔvacA* for 24 and 48 h **(A)** *MICA* and *MICB* mRNA levels were determined by qPCR. **(B)** Soluble MICA and MICB levels in cell culture supernatants were determined by ELISA. **(C–E)** AGS-MICA cells were challenged with *H. pylori* WT and isogenic mutants *ΔcagA*, *ΔcagL* and *ΔvacA* for 24 h **(C)** *MICA* mRNA levels were determined by qPCR. **(D)** MICA protein in cell lysates was determined by Western Blot. **(E)** Soluble MICA protein in cell culture supernatants was determined by ELISA. Experiments were performed three times. Mean ± SD, one-way ANOVA and Tukey’s test (ns, not significant, *P <0.05; **P <0.01; ***P <0.001; ****P <0.0001).

Cell culture supernatants were passed through 0.22 µM sterile filters and were concentrated with Amicon^®^ Ultra-2 mL Centrifugal Filters (MerckMillipore), according to the manufacturer’s instructions. We applied the same starting volume (2 ml) of all culture supernatants to the Amicon^®^ Ultra-2 mL Centrifugal Filters and centrifuged until all samples were reduced to less than 500 µl. Because the procedure reduced all samples to slightly different volumes, we then added varying amounts of fresh reagent diluent to the samples, to adjust all samples to the equal volume of 500 µl. To take this 4-fold concentration into account, we divided the ELISA-readout values by 4 and reported these final values in **Figures 2**, **3**. ELISA was performed using the Human MICA Duoset ELISA kit (R&D) and the Human MICB Duoset ELISA kit (R&D), according to the manufacturers protocol. Readouts were performed on a SPECTROstar Omega Plate reader.

### 2.8 Transfection of AGS cells

For the generation of AGS cells expressing the MICA allele *019 (A5) (denoted AGS-MICA cells), we used the lentiviral vector pHRSIN (41), a gift of Prof. Paul Lehner (Cambridge Institute for Medical Research, Cambridge, United Kingdom), expressing MICA*019 under control of the spleen focus-forming virus (SFFV) promoter for constitutive, high-level gene expression. MICA*019 encodes a full-length MICA protein with all the features that are typical for these allelic variants (42). Lentiviruses were generated by means of transfection of 293T cells with pHRSIN, together with the plasmids pCMVR8.91 and pMD2G. Two days after transfection, culture media containing the lentiviruses were harvested, filtered, and stored at −80°C. For each lentiviral transduction, 0.3 × 10^6^ AGS cells were mixed with 0.75 mL of virus supernatant in the presence of 1 μmol/L of the TBK1 inhibitor BX795 (InvivoGen) and 8 μg/mL Polybrene (Sigma-Aldrich) and seeded into one well of a 6-well plate (BD Biosciences). The plates were centrifuged at 800 rpm at 33°C for 1 h. After centrifugation, without removing viral supernatants, the plates were incubated at 37°C in a 5% CO2 atmosphere for 4-6 h and then centrifuged again with the same conditions. Supernatants were removed from wells, and fresh growth medium was added. After two weeks of cultivation, AGS-MICA transfectants were stained with the Alexa Fluor^®^ 647 anti-human MICA/MICB Antibody (BioLegend Cat# 320914, RRID: AB_2266419) and sorted by FACS.

### 2.9 Western blot

Protein lysates from cell cultures were prepared in RIPA buffer (Merck Millipore) containing 0.1 mmol/L Pefabloc, 1 mmol/L DTT, cOmplete Mini EDTA-free and PhosSTOP (Roche). Protein concentrations were determined using the BioRad Protein Assay Dye Reagent (BioRad Laboratories). Proteins were loaded onto 10% (v/v) (SDS)-polyacrylamide gels, subjected to electrophoresis and then blotted onto PVDF membranes (Immobilon-P, Merck Millipore). Blotting efficiency was determined by staining with Ponceau S solution (Sigma Aldrich). Non-specific binding was blocked for 1 h with 5% (w/v) non-fat dry milk (Bio-Rad Laboratories) in tris-buffered saline (TBS) with 0.1% (v/v) Tween 20 (Merck Millipore). Subsequently, the membranes were incubated with the primary antibody MICA biotinylated antibody (R&D Systems Cat# BAF1300, RRID: AB_355943, 1:2000) overnight at 4°C, followed by incubation with streptavidin-HRP (R&D Systems DY998, 1:5000) at RT for 1 h. For loading control, membranes were incubated with GAPDH antibody (Cell Signaling Technology #2118, 1:1000), overnight at 4°C, followed by incubation with rabbit IgG HRP linked F(ab′)2 (Merck, GENA9340-1ML, 1:5000) at RT for 1 h. Immunolabeling was detected using the ECL™ Select Western Blotting Detection Reagent (Merck, GERPN2235) and visualized with the ImageQuant™ LAS 500. Quantification of band intensities was performed using Image Lab Software (Bio-Rad).

### 2.10 Flow cytometry

Cells were harvested with Accutase^®^ (Thermo Fisher Scientific) and stained with Alexa Fluor 647 anti-human MICA/B antibody (BioLegend, Cat# 320914, RRID: AB_2266419) at 4°C for 1 h. Flow cytometry was performed using a CytoFLEX S flow cytometer (Beckman Coulter) according to the manufacturer’s protocol. The software CytExpert (Beckman Coulter) was used for the analysis of flow cytometry data. Flow cytometry results are represented as median fluorescence intensity (MFI).

### 2.11 Immunofluorescence

Cells were grown on adhesive slides (QPath) with flexiPERM^®^ (Sarstedt) and infection assays were performed as described above. Subsequently, cells were fixed in 4% formaldehyde (Thermo Scientific #28906) and permeabilized with 0,1% saponin before incubation with the Alexa Fluor^®^ 647 anti-human MICA/MICB antibody (BioLegend Cat# 320914, RRID: AB_2266419, 1:100). 4’,6-Diamidin-2-phenylindol (DAPI) was used for nuclear counter-staining. Fluorescence staining was analyzed using the Zeiss LSM510 Meta Confocal Microscope. The Nikon software NIS-Elements General Analysis (GA3) was used for quantification of fluorescence intensity per cell.

### 2.12 MICA genotyping

Genotyping of the microsatellite repeat polymorphism in the transmembrane region of the *MICA* gene, was carried out as described (43). Fragment sizes were determined using the Peak Scanner v1.0 software (Applied Biosystems). The cell lines Jurkat (A5.1/A6), J82 (A5.1/A6), RT4 (A5.1) and RT112 (A4) were used as controls.

### 2.13 Analysis of NKG2D expression on NKL cells

AGS-MICA cells were either not infected or infected with *H. pylori*, as described above. After 24 h, cell culture supernatants were harvested, passed through 0.22 µM sterile filters and concentrated with centrifugal filters (Amicon Ultra-15, PLGC Ultracel-PL Membran, 10 kDa, Merck Millipore). Cell culture supernatants were concentrated 10-fold and then diluted with fresh RPMI medium by 2 (to generate 5-fold concentrated supernatants) and by 5 (to generate 2-fold concentrated supernatants). Next, we added 50 µl of these supernatants (10-fold, 5-fold and 2-fold concentrated) to 50 µl of NKL cells per well, resulting in a further 2-fold dilution and thus a final treatment of NKL cells with 5-fold, 2,5-fold and 1-fold concentrated supernatants. NKL cells were treated with these cell culture supernatants in the presence of 5 µg/ml of the human MICA/B antibody (R&D Systems Cat# MAB13001, RRID: AB_2143621) or the mouse IgG2A isotype control (R&D Systems Cat# MAB003, RRID: AB_357345). After 24 h, NKL cells were stained either with the FITC anti-human CD94 antibody (BioLegend Cat# 305504, RRID: AB_314534) or the human NKG2D/CD314 antibody (R&D Systems Cat# MAB139, RRID: AB_2133263), followed by staining with the goat F(ab’)2 anti-mouse IgG - (Fab)’2 (PE), pre-adsorbed (Abcam Cat# ab5889, RRID: AB_955482) at 4°C for 30 min. Flow cytometry readouts are represented as median fluorescence intensity (MFI).

### 2.14 Analysis of cytotoxic degranulation of NK cells

NKL cells were treated with cell culture supernatants from AGS-MICA cells, as described above. Subsequently, NKL cells were co-cultured with K562 cells at 37°C for 2 h. The cultures were then stained with the FITC anti-human CD94 antibody (BioLegend Cat# 305504, RRID: AB_314534) and the APC anti-human CD107a (LAMP-1) antibody (BioLegend Cat# 328620, RRID: AB_1279055) at 4°C for 30 min. Flow cytometry readouts are represented as % of the LAMP-1+ cells, out of the CD94+ cells.

### 2.15 Statistical analysis

For cell culture assays, data from three independent experiments were combined and parametric tests were performed to determine statistically significant differences between groups. One-way analysis of variance (ANOVA) was used to compare three or more groups with Dunnett’s multiple comparisons test to compare the mean of each group with the mean of one single control group or with Tukey’s multiple comparisons test to compare the mean of each group to the mean of every other group. An unpaired, two-tailed Student’s t-test was used to compare two independent groups and a one-sample t-test was used to compare the mean of one group to a theoretical mean of 1. Data from patient biopsies were tested for normality using the Shapiro-Wilk test. Subsequently, the data were subjected to statistical analysis by t-test or ANOVA for normally distributed sample sets, or to analysis by Kruskal Wallis test for nonparametric analyses. As *post-hoc* test, Dunn’s multiple comparisons test was applied to compare the mean rank of each group with the mean rank of every other group. A value of P<0.05 was considered significant (*P <0.05; **P <0.01; ***P <0.001; ****P <0.0001). Statistical analyses were performed with GraphPad Prism 9.

## 3 Results

### 3.1 The NKG2D system is altered in *H. pylori* infection and gastric cancer

*H. pylori* elicits a strong inflammation of the stomach mucosa, but the contribution of effector cells harboring the NKG2D receptor, like CTLs and NK cells, within the immune infiltrate is poorly described (44). We immuno-phenotyped biopsies from healthy controls (Healthy), *H. pylori* gastritis cases (HpG) and gastric adenocarcinoma cases (Cancer) by immunohistochemistry (IHC) (**Figures 1A, B**). HpG demonstrated substantially higher numbers of leukocytes (pan-leucocyte marker CD45) in the mucosa compared to healthy controls. Leucocytes were also significantly higher in cancer compared to heathy controls (**Figures 1A, B**). However, the numbers of CTLs (CD8) and NK cells (CD56, NKp46) were unchanged in HpG compared to healthy controls. In cancer, CTLs (CD8) were significantly lower while NK cells (CD56, NKp46) were significantly higher compared to healthy controls (**Figures 1A, B**). Overall, only sparse NK cell signals were detectable in HpG and controls with the NK cell markers CD56 and NKp46. Gene expression analysis of additional NK cell marker genes confirmed the observed lack of NK cell induction in HpG (**Supplementary Figure 3**). Next, we assessed expression of NKG2D system genes (*NKG2D, MICA* and *MICB*) via qPCR (**Figure 1C**). *NKG2D* receptor expression was significantly lower in HpG compared to healthy controls and even more so in cancer (**Figure 1C**). Expression of the ligand *MICA* was not changed in HpG but was lower in cancer compared to healthy controls. Expression of *MICB* was significantly higher in both conditions compared to healthy controls (**Figure 1C**). There were no significant differences in expression of lymphocyte markers and NKG2D system genes in relation to gender (data not shown). In summary, despite strong overall immune activation in *H. pylori*-associated pathologies, the numbers of CTLs and NK cells do not appear to be considerably changed in gastritis compared to the healthy state. Importantly, expression of the *NKG2D* receptor is significantly diminished and the NKG2D-L *MICB* is induced in both conditions. As most cancer samples were *H. pylori*-negative, the dysregulation of *NKG2D* and *MICB* was likely due to cancer-induced immune evasion. However, in HpG, the observed modulations of these genes suggest that *H. pylori* manipulates the NKG2D system.

### 3.2 *H. pylori*-dependent modulation of NKG2D-L expression and soluble release via proteolytic shedding

NKG2D evasion typically occurs either through repression of NKG2D-L expression or by the release of soluble NKG2D-Ls from the cell surface (17, 18). To determine a potential role of *H. pylori* in the modulation of *MICA* and *MICB* gene expression, we infected gastric epithelial cell line MKN28 with the *H. pylori* P12 wild-type (WT) strain. Treatment with *Cutibacterium acnes (C. acnes)* and the SCFA butyrate, both previously shown to induce NKG2D-L expression, served as controls (26, 39). After 24 and 48 h of treatment, *MICA* and *MICB* mRNA expression was determined by qPCR (**Figure 2A**). Butyrate induced both genes compared to non-infected cells, while *C. acnes* substantially affected only MICB (**Figure 2A**), consistent with previous findings (26, 39). Importantly, *H. pylori* WT infection resulted in significantly increased *MICA* and *MICB* mRNA levels compared to non-infected cells, and induction increased over time (**Figure 2A**). *MICB* was more induced than *MICA* and the degree of inducibility seemed to correlate negatively with the level of baseline mRNA expression of *MICA* and *MICB* in these cell lines (**Supplementary Figure 4**). *H. pylori* infection also upregulated gene expression of the NKG2D activator interleukin 15 (*IL-15*), the NKG2D ligands *ULPB1* and *ULBP2* and the NKG2D inhibitor transforming growth factor beta (*TGF-β*) compared to non-infected cells, whereas the NKG2D inhibitor macrophage migration inhibitory factor (*MIF*) was not induced (**Supplementary Figures 5, 6**) (45, 46).

We next asked whether *H. pylori* infection affects the release of soluble NKG2D-Ls. To determine this, we analyzed levels of soluble MICA (sMICA) and soluble MICB (sMICB) in cell culture supernatants of infected MKN28 cells via ELISA (**Figure 2B**). Butyrate induced sMICA and sMICB levels compared to non-infected cells, especially at 48 h, while *C. acnes* did not (**Figure 2B**), consistent with the notion that this bacterium activates the NKG2D system rather than evading it (26). Importantly, *H. pylori* WT infection significantly increased sMICA and sMICB compared to non-infected cells, especially at 48 h (**Figure 2B**). sMICB was also significantly increased in gastric epithelial cell line AGS after *H. pylori* infection, despite only minor induction of *MICB* gene expression in this cell line (**Supplementary Figure 7**). No sMICA was detected in the supernatants of AGS cells, since this cell line is MICA protein-deficient due to a single amino acid substitution (46) that interferes with protein folding (47).

We then aimed to elucidate the mode of soluble release of sMICA and sMICB. NKG2D-Ls can be released from the cell surface, either by proteolytic shedding or via extracellular vesicles (EVs), depending on the cell type, allele and treatment conditions (**Supplementary Figure 8**) (48, 49) Genotyping of our cell lines showed them to be homozygous for MICA alleles A5 (AGS) and A6 (MKN28), both of which amenable to proteolytic shedding. Regarding MICB, no alleles associated with extracellular vesicles release are known. Extracellular vesicle-enriched preparations from supernatants of *H. pylori*-infected cells showed only negligible amounts of MICA/B protein compared to extracellular vesicle-free supernatants, suggesting that there is no relevant extracellular vesicle release of MICA/B in these cell lines (**Supplementary Figure 9**). Next, we treated MKN28 and AGS cells with *H. pylori* WT or butyrate, to induce soluble release of MICA/B, and simultaneously added the broad-spectrum metalloprotease inhibitor batimastat dissolved in DMSO (40). As solvent control we only added DMSO. After 48 h of treatment, we assessed sMICA and sMICB by ELISA (**Figure 2C**). Batimastat significantly impaired the release of sMICA and sMICB from MKN28 cells and sMICB from AGS cells (**Figure 2C**). This indicates that *H. pylori-* and butyrate-induced release of sMICA/B, as well as its constitutive release is mediated by metalloproteases that can be specifically blocked (**Figure 2C**). Silencing of *ADAM17* and *MMP9*, two proteases previously associated with proteolytic shedding of MICA/B (40, 50–53), had no significant impact on shedding frequencies (**Supplementary results and Figure 10**). Thus, the specific metalloproteases responsible for MICA/B shedding in *H. pylori* infection remain to be determined. Taken together, *H. pylori* WT infection induces gene expression and proteolytic shedding of NKG2D-Ls in stomach epithelial cells, suggesting evasion of the NKG2D system.

### 3.3 *H. pylori* virulence factor-dependent modulation of NKG2D-L expression and soluble release

To determine a potential role of the major *H. pylori* virulence factors in the modulation of NKG2D-Ls, we infected MKN28 cells with isogenic mutants of cytotoxin-associated gene A (*ΔcagA*), cytotoxin-associated gene L (*ΔcagL*) and vacuolating cytotoxin A (*ΔvacA*) (3). Infection with the *ΔcagA* and *ΔcagL* mutants resulted in only slightly lower levels of *MICA* and *MICB* mRNA expression, whereas infection with the *ΔvacA* mutant resulted in greatly lower levels of *MICA* and *MICB* mRNA expression, compared to infection with the WT (**Figure 3A**). Thus, *vacA* appears to be particularly important for inducing *NKG2D-L* mRNA expression. Regarding soluble NKG2D-Ls, infection with all three mutants (*ΔcagA*, *ΔcagL* and *ΔvacA*) resulted in lower sMICA levels and infection with the *ΔcagA* and *ΔcagL* mutants resulted in significantly lower sMICB levels compared to infection with the WT at 48 h (**Figure 3B**). We then speculated that *cagA* and *cagL* might be important for inducing the soluble release of NKG2D-Ls. Notably, cells infected with the *ΔvacA* mutant showed increasing sMICB levels from 24 h to 48 h without any increase in *MICB* mRNA levels. They also showed similar sMICB levels compared to cells infected with the *ΔcagA* and *ΔcagL* mutants, despite significantly lower *MICB* mRNA levels. Based on these observations, we hypothesized, that *H. pylori* might induce the release of soluble NKG2D-Ls *independent* of its effect on NKG2D-L gene expression.To specifically study the effect of *H. pylori* on the soluble release of NKG2D-Ls *independent* of its effect on NKG2D-L gene expression, we aimed to use a cell line where NKG2D-L gene and protein expression is unaffected by *H. pylori*. For this purpose, we used the cell line AGS where *MICA* mRNA expression was unaffected by *H. pylori* infection (**Supplementary Figure 7A**) and which is MICA protein-deficient (**Supplementary Figure 7B**) (47). To achieve constitutive MICA protein expression that is unlikely to be affected by *H. pylori*, we transfected AGS cells with a *MICA*-overexpression construct driven by the spleen focus forming virus (SFFV) promoter (the transfectants were termed AGS-MICA). As expected, infection of AGS-MICA transfectants with *H. pylori* WT or the *ΔcagA, ΔcagL* and *ΔvacA* mutants had no significant impact on total *MICA* mRNA expression (**Figure 3C**) and total MICA protein levels in whole cell lysates (**Figure 3D**; **Supplementary Figure 11**). Importantly, sMICA levels in cell culture supernatants of AGS-MICA cells significantly increased after challenge with *H. pylori* WT compared to non-infected cells (**Figure 3E**), indicating that *H. pylori* induces the release of soluble MICA, even when MICA gene and protein expression is unaffected. An increase in sMICA was also seen after infection with the *ΔvacA* strain but not with the *ΔcagA* and *ΔcagL* strains compared to non-infected cells (**Figure 3E**). Taken together, induction of *MICA/B* gene expression seems to be a *vacA*-dependent process, while soluble release of MICA appears to be *cagA/L*-dependent. Combined, these virulence factor-dependent effects could result in the release of large amounts of soluble NKG2D-Ls, while epithelial cells might have reduced levels of NKG2D-Ls at the cell surface, protecting them from immunosurveillance.

### 3.4 Translocation of NKG2D-Ls upon *H. pylori* infection

**Figure 4:**
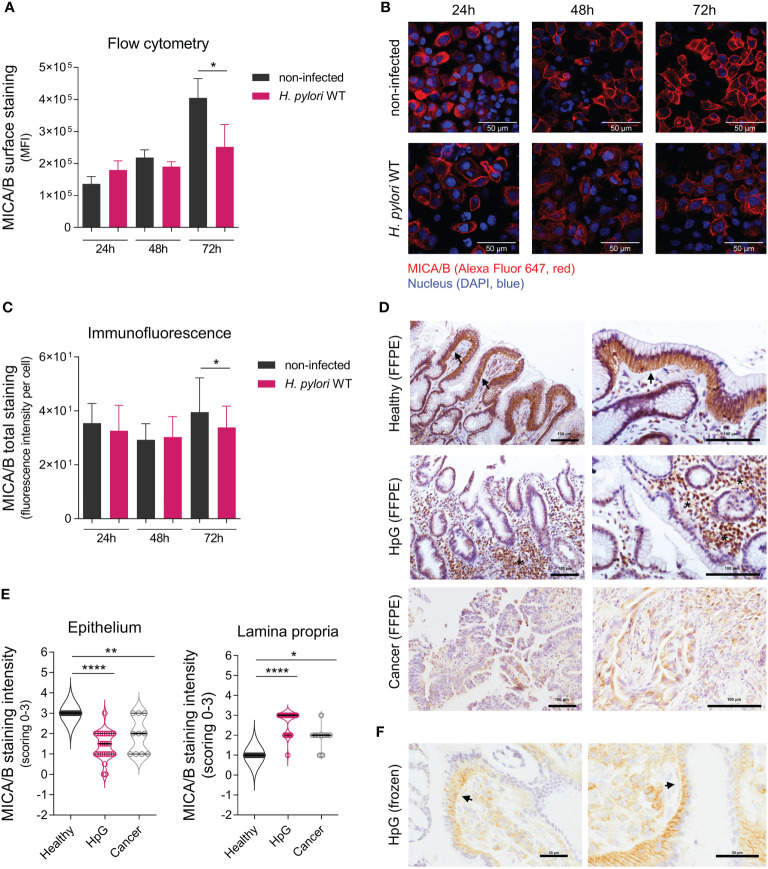
Translocation of NKG2D-Ls upon *H. pylori* infection. AGS-MICA cells were infected with *H. pylori* WT for 24, 48 and 72 h **(A)** MICA/B cell surface expression was quantified by flow cytometry. Experiments were performed three times. **(B, C)** Total cellular MICA/B protein in permeabilized cells was visualized by immunofluorescence (IF) staining with an anti-MICA/B antibody (red) and DAPI (blue). **(B)** Representative images, scale bars: 50µm. **(C)** Quantification of MICA/B staining-intensity per cell in IF micrographs. Experiments were performed six times. The data shown in a and c are represented as mean ± SD, unpaired, two-tailed Student’s t-test of infected versus non-infected cells at each timepoint (*P <0.05). **(D, E)** IHC staining of MICA/B in stomach biopsies (FFPE) from healthy controls (Healthy), *H. pylori* gastritis cases (HpG) and stomach adenocarcinoma cases (Cancer). **(D)** Representative images with arrows indicating basolateral epithelial staining and stars indicating lamina propria staining, scale bars: 100µm. **(E)** Quantification of MICA/B in biopsies by scoring of epithelial and lamina propria staining intensities from 0 (= no staining) to 3 (= strong staining), n=10-27 per group, the data do not follow normal distribution, violin plots (median, quartiles and all points), Kruskal-Wallis test and Dunn’s multiple comparisons test (*P <0.05; **P <0.01; ****P <0.0001). **(F)** Staining of frozen HpG biopsies, showing a distinct subepithelial MICA/B deposition (arrows), scale bars: 50µm.

To determine the effect of *H. pylori* on the cell surface expression of NKG2D-Ls, we measured cell surface MICA/B proteins by flow cytometry (**Figure 4A**). In non-infected AGS-MICA cells, cell-surface MICA/B staining increased with time, likely due to continuous MICA/B production in these cells (**Figure 4A**). Cells infected with *H. pylori* showed significantly lower MICA/B surface staining compared to non-infected cells after 72 h, possibly due to a continuous release of soluble MICA/B from the cell surface (**Figure 4A**). Visualization with immunofluorescence confirmed these findings, wherein *H. pylori*-infected cells showed significantly less MICA/B staining compared to non-infected cells after 72 h (**Figures 4B, C**).

We next assessed MICA/B protein localization in human stomach biopsies by IHC (**Figures 4D–F**). In healthy controls, strong intracellular MICA/B staining was observed in epithelial cells (**Figures 4D, E**), consistent with the presumed intracellular MICA/B protein localization (11, 12), and likely indicating the cells’ constitutive readiness to respond to stress. Only sparse MICA/B staining was detectable in the lamina propria in healthy controls (**Figures 4D, E**). In HpG, epithelial MICA/B staining was significantly lower compared to healthy controls, or completely absent (**Figures 4D, E**). However, abundant MICA/B staining was visible in the lamina propria of HpG cases, which was either associated with the immune infiltrate or free without any cell association (**Figures 4D, E**). MICA/B staining in HpG was also visible as a rim below the epithelial cells (**Figure 4F**), suggesting that epithelial MICA/B was translocated from the basolateral site to the lamina propria. In stomach cancer, epithelial MICA/B staining was also lower and lamina propria staining was also higher compared to healthy controls, but not as high as in HpG (**Figures 4D, E**). There were no significant differences in MICA/B expressions in relation to gender (data not shown). Altogether, these data suggest that in HpG and gastric adenocarcinoma, a translocation of NKG2D-Ls from the epithelia to the lamina propria likely happens. Consequently, soluble NKG2D-Ls in the lamina propria could encounter NKG2D-expressing effector cells and attenuate their cytotoxicity.

### 3.5 Soluble NKG2D-Ls from *H. pylori*-infected epithelia reduce surface expression of the NKG2D receptor and cytotoxic degranulation of NK cells

**Figure 5:**
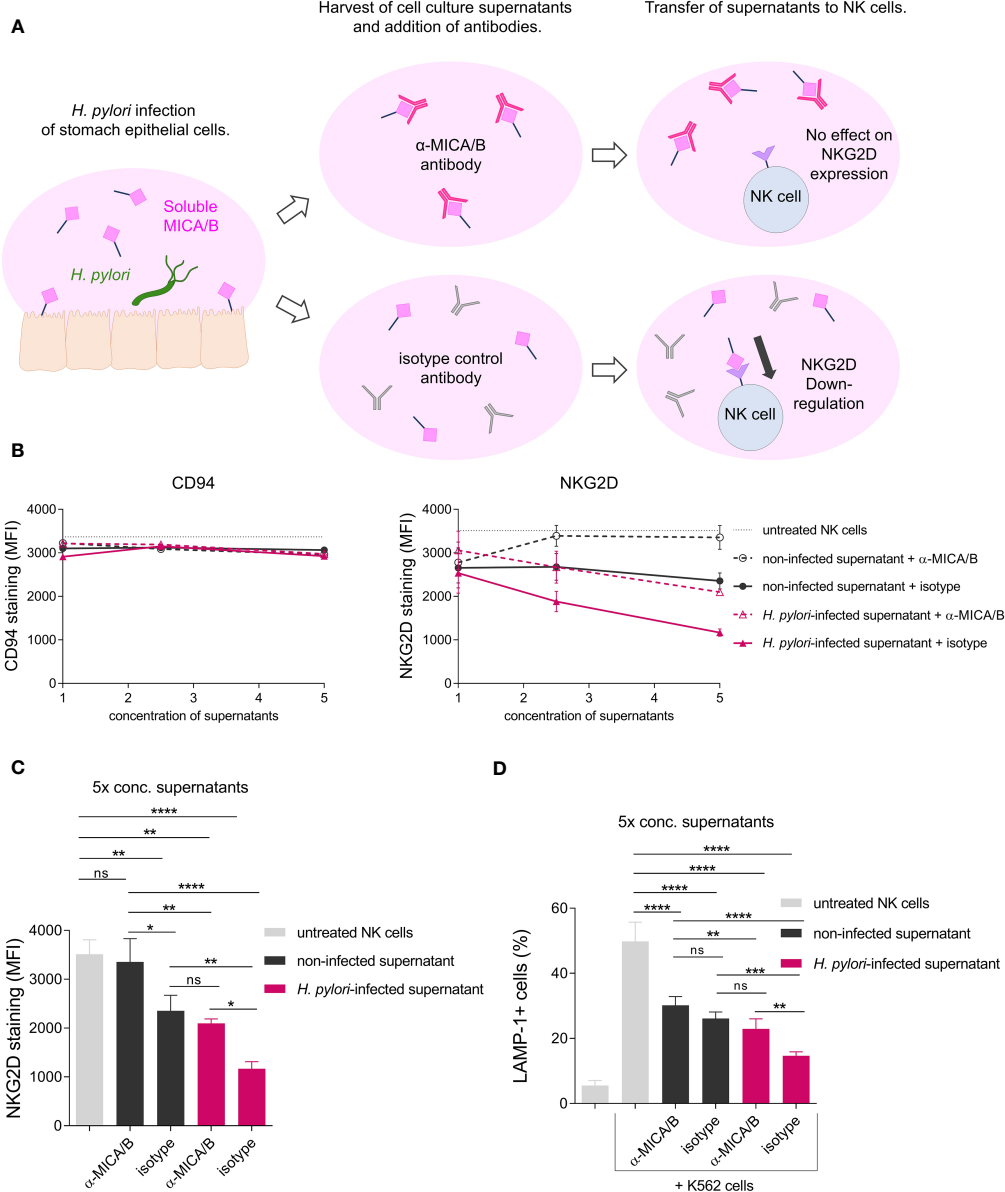
Soluble NKG2D-Ls from *H. pylori*-infected epithelia reduce surface expression of the NKG2D receptor and cytotoxic degranulation of NK cells. **(A)** Scheme of the experimental setup. **(B, C)** The NK cell line NKL was challenged with filter-sterilized cell culture supernatants from non-infected AGS-MICA cells (‘non-infected supernatant’) or from *H. pylori*-infected AGS-MICA cells (‘*H. pylori*-infected supernatant’). Supernatants were filter-concentrated and applied at three concentrations (1x, 2.5x, 5x) and either a neutralizing anti-MICA/B antibody (‘α-MICA/B’) or an isotype control antibody (‘isotype’) were added to the cultures. After 24 h, cell surface expressions of CD94 and NKG2D and were analyzed by flow cytometry. NKG2D expression after treatment with 5x concentrated supernatants is individually shown in **(C, D)** NKL cells were treated for 24 h with 5x concentrated supernatants as described above and then co-cultivated with K562 cells for 2 h Percentages of LAMP-1+ cells (from CD94+ cells) were determined by flow cytometry. Experiments were performed three times, mean ± SD. One-way ANOVA and Tukey’s test (*P <0.05; **P <0.01; ***P <0.001, ns, not significant).

Soluble NKG2D-Ls act as strong repressors of NKG2D-mediated immunity by reducing NKG2D expression of effector cells and thereby diminishing NKG2D-mediated effector cell cytotoxicity (20–22). To determine the effect of *H. pylori*-induced soluble MICA/B on the expression of NKG2D on effector cells, we challenged NK cells (NKL cell line) with filter-sterilized cell culture supernatants from *H. pylori*-infected or non-infected AGS-MICA cells (termed ‘*H. pylori*-infected supernatant’ and ‘non-infected supernatant’). Supernatants were filter-concentrated and applied at three concentrations (1x, 2.5x and 5x concentrated). To neutralize the effects of sMICA/B, an anti-MICA/B neutralizing antibody (termed ‘α-MICA/B’), was added to the cultures. Alternatively, an isotype control antibody (termed ‘isotype’) was used. After 24 h of challenge, cell surface expressions of NK cell proteins were analyzed by flow cytometry (**Figures 5A–C**). Control measurements of the NK cell surface marker CD94 showed no changes after any treatment, ruling out non-specific effects of the supernatants on the expression of NK cell surface proteins (**Figure 5B**). NKG2D expression was reduced by treatment with *H. pylori*-infected supernatant + isotype compared to untreated NK cells, and the reduction was concentration-dependent, with the greatest reduction being induced by 5x concentrated supernatant (**Figures 5B, C**). Treatment with non-infected supernatant + isotype also reduced NKG2D surface expression compared to untreated NK cells, although to a lesser extent compared to *H. pylori*-infected supernatant + isotype (**Figures 5B, C**), consistent with the constitutive shedding of NKG2D-Ls from AGS-MICA cells (**Figure 3E**). Importantly, addition of the anti-MICA/B antibody significantly attenuated NKG2D downregulation (**Figures 5B, C**), indicating that sMICA/B proteins in the supernatants cause the reduction of NKG2D expression. The effect of *H. pylori*-infected supernatant was not completely abrogated by addition of the anti-MICA/B antibody (**Figures 5B, C**), suggesting that additional factors in these supernatants also contribute to NKG2D downregulation.

**Figure 6:**
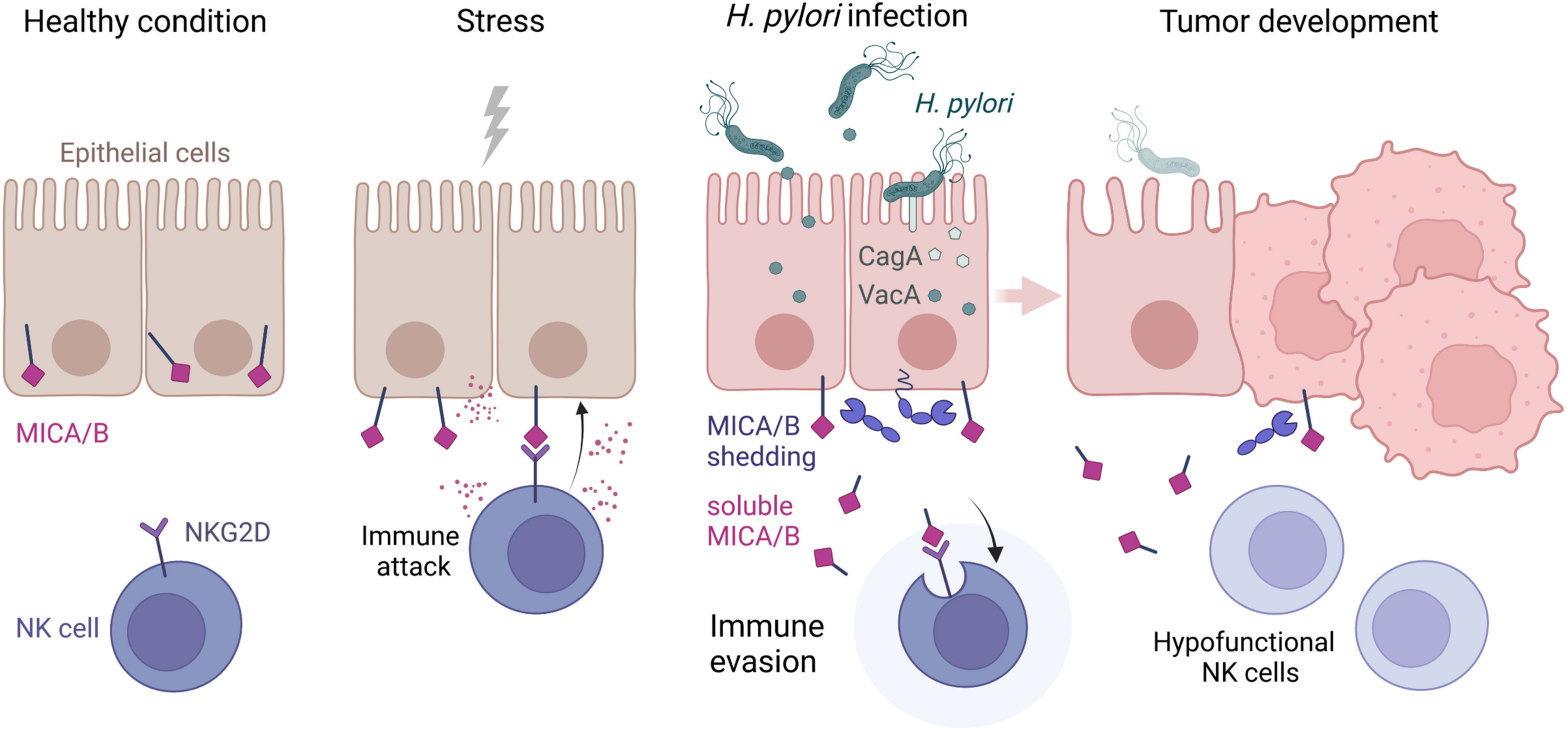
Scheme highlighting the NKG2D system modulation by *H. pylori*. Healthy epithelia store MICA and MICB proteins intracellularly. Upon stress, MICA/B are expressed at the cell surface and bound by the immunoreceptor NKG2D, which activates immune attack by NKG2D-harboring effector cells. During *H. pylori* infection, virulence factors CagA and VacA modify the expression and proteolytic shedding of MICA/B. Soluble MICA/B proteins lead to immune evasion by binding NKG2D, which results in internalization and downregulation of NKG2D and a suppression of lymphocyte cytotoxicity. Hypofunctional NK and cytotoxic T cells in the stomach lamina propria could allow transformed cells to escape immune surveillance and facilitate tumor development. Scheme drawn with BioRender (https://biorender.com).

To characterize the functional consequence of reduced NKG2D expression, we co-cultured NKL cells with the tumor cell line K562 for 2 h and then measured cell surface expression of lysosomal-associated membrane protein 1 (LAMP-1) on NKL cells as a marker for cytotoxic degranulation, indicating the cytotoxic reactivity of NK cells toward tumor cells (54). Co-cultivation with K562 cells induced a marked increase in LAMP-1 expression on untreated NKL cells (**Figure 5D**), confirming activation of NK cell cytotoxic degranulation upon exposure to tumor cells. Next, we pre-treated NKL cells with cell culture supernatants from *H. pylori*-infected or non-infected AGS-MICA cells as described above, before co-cultivation with K562 cells. NKL cells pre-treated with *H. pylori*-infected supernatant + isotype showed significantly reduced LAMP-1 expression compared to untreated NKL cells (**Figure 5D**). This reduction was significantly reversed by addition of the anti-MICA/B antibody during the pre-treatment (**Figure 5D**), indicating that sMICA/B proteins in *H. pylori*-infected supernatants act to suppress NK cell cytotoxic degranulation. NKL cells pre-treated with non-infected supernatant + isotype also showed reduced LAMP-1 expression compared to untreated NKL cells, but to a lesser extent compared to NKL cells pre-treated with *H. pylori*-infected supernatant + isotype (**Figure 5D**). This reduction was not reversed by addition of the anti-MICA/B antibody (**Figure 5D**), thus, it was not driven by MICA/B but likely by other immunomodulatory factors secreted by these stomach epithelial cells. Notably, tumor cell killing was also diminished when NKL cells were pre-treated with supernatants from *H. pylori*-infected epithelial cells, compared to untreated NKL cells or NK cells pre-treated with supernatants from non-infected epithelial cells (**Supplementary Figure 13**). In summary, these data demonstrate that *H. pylori* infection induces the release of soluble NKG2D-Ls from stomach epithelial cells which leads to reduced NKG2D receptor expression and attenuated cytotoxic degranulation of NK cells. Thus, impairment of the NKG2D system by *H. pylori* may weaken immune surveillance which could facilitate the development of gastric cancer (**Figure 6**).

## 4 Discussion

The immune activating receptor NKG2D and its ligands represent an immune recognition system important for the activation of NK cells, cytotoxic- and γδ T-cells in response to cellular stress such as infection or oncogenic transformation (6). The NKG2D system is involved in mucosal immunity (24), and altered in gastrointestinal pathologies such as gastritis, celiac disease and Crohn’s disease (26–28). It also represents a key component for tumor immune surveillance and tumors as well as viruses have acquired mechanisms to escape NKG2D-dependent immunity (17). Using human tissues and cell culture-based infection models we show that the NKG2D system can be influenced by *H. pylori*. Induction of NKG2D-L expression and the release of these ligands as soluble molecules were *H. pylori* virulence factor-dependent processes and mucosal NKG2D-L deposition was evident in *H. pylori*-associated pathologies. By modeling the effect of soluble NKG2D-Ls on effector cells, we verified *H. pylori’s* ability to attenuate NK cytotoxic activity, providing evidence for a so far unprecedented immune evasion mechanism by this prominent human pathogen.

Gastric *H. pylori* infection is characterized by pronounced inflammation of the mucosa, exhibiting abundant infiltration with plasma-, B- and T-cells with pro- (e.g., T helper cells Th1, Th17) but also anti- (e.g., Tregs) inflammatory properties. Individual compositions of these leucocytes influence disease courses (44, 55). The role of CTLs and NK cells in the stomach in *H. pylori* negative and positive individuals is less defined (44), and variable quantities of these cell types have been reported (56–59). We found no induction of NK cell and CTL numbers by IHC, despite a strong general immune infiltration in *H. pylori* gastritis. NKG2D is one of the main activating receptors of NK cells and a major co-stimulatory receptor of CTLs (6). We observed a significant downregulation of *NKG2D* gene expression by qPCR. In parallel, the NKG2D-L *MICB* was significantly upregulated in gastritis and adenocarcinoma cases, suggesting a dysregulation of the NKG2D system. Besides NKG2D-Ls (60) certain cytokines might also regulate NKG2D receptor expression, like IL-15 acting as an inducer or TGF-β and MIF acting as repressors (45, 46). We found that *H. pylori* infection induced *IL-15* and *TGF-β* in AGS and MKN28 cells whereas *MIF* was not induced. Since these cytokines could also be produced by other cell types including leukocytes or stromal cells in addition to epithelial cells, various cell types might influence NKG2D system dysregulation in different gastric pathologies.

NKG2D-Ls are generally absent on cell surfaces in health (12). In the gut, their expression is also co-regulated by the microbiota (25). Stress conditions such as infections or neoplastic transformation, however, induce their *de novo* expression and surface display (8, 13–15). This induction of surface expression can be overcome by viruses and tumors, either by downregulating NKG2D-L expression or by releasing NKG2D-Ls as soluble proteins from the cell surface (17–19, 61, 62). Soluble NKG2D-Ls attenuate the strength of the immune response by acting as suppressors of NKG2D-expressing effector cells. Their binding to the receptor leads to NKG2D downregulation and thus diminished NKG2D-mediated immunity, as well as a general impairment of effector cell function (20–23). Using cell-culture based infection models, we found that *H. pylori* infection of stomach epithelial cells induced both gene expression and soluble release of the NKG2D-Ls MICA and MICB and reduced MICA/B cell surface expression. *In vivo*, MICA/B was reduced at epithelial cells but accumulated in the lamina propria in gastritis and adenocarcinoma cases. Since NK cells are essential for early detection of carcinogenesis (63), soluble NKG2D-Ls in the lamina propria in gastritis might impair the eradication of spontaneously transformed cells, thereby facilitating cancer development. In gastric adenocarcinoma, the elevated MICA/B levels in the lamina propria were likely a consequence of immune evasion by the cancer itself, since *H. pylori* is usually lost from the stomach with the development of advanced disease (64), and undetectable by the time of clinical cancer diagnosis. Release of soluble NKG2D-Ls is common in advanced cancer and high serum levels of soluble NKG2D-Ls correlate with a systemic reduction of NKG2D expression and with high cancer stage, metastatic disease and overall poor prognosis (20, 65–67). Therapeutic applications to overcome NKG2D system evasion in cancer could be a useful complement to other forms of tumor immunotherapy and are currently being explored (18). In our study we focused on NK cells, as they are the most extensively studied cell type in the context of the NKG2D-mediated immune response. Other NKG2D-expressing cell types like γδ T cells were also shown to play important roles in tumor immunity (15, 68). γδ T cells are highly abundant in the gut mucosa as intraepithelial lymphocytes (IELs) where they are important for immune surveillance and tissue homeostasis (69). Thus, inhibition of γδ T cells by *H. pylori* could potentially also contribute to bacterial persistence and the promotion of neoplastic processes.

*H. pylori* strains differ in their allelic compositions of the main virulence factors *vacA* and *cagA*. VacA is a pore-forming toxin and all strains harbor the *vacA* gene but different alleles exist wherein the alleles s1, m1 and i1 are associated with a higher risk for stomach cancer (1). CagA interferes with various host cell signaling processes and the *cagA* gene is considered an oncogene (70). CagA is injected into host cells by a type IV secretion system (T4SS), where the protein CagL is a crucial component for CagA translocation. Not all strains carry *cagA* and different *cagA* alleles vary in their virulence potential (71). Interestingly, *cagA* and *vacA* alleles show genetic associations. C*agA* positive strains typically contain the more pathogenic *vacA* alleles and the two toxins seem to counterbalance each other’s effects to reduce mucosal harm, allowing persistent colonization of *H. pylori* (1, 37). We identified another example for this synergism. While *vacA* seemed to induce *NKG2D-L* gene expression, which could activate the immune response against infected epithelial cells, *cagA* seemed to trigger NKG2D-L shedding, which can reduce the cells’ immunogenic visibility. The combined actions of both toxins might potentiate the amount of soluble NKG2D-Ls released, which could result in strong suppression of NKG2D-mediated immunity. The *H. pylori* strain P12 used in this study, which showed *vacA*-dependent induction of *NKG2D-L* gene expression, harbors the highly pathogenic *vacA* allele s1m1 (33). Notably, induction of *NKG2D-L* gene expression was absent in our previous study, where we used the *H. pylori* strains PMSS1 and SS1, which contain the less pathogenic *vacA* allele s2m2 (26, 72). Thus, induction of *NKG2D-L* gene expression is likely associated with specific *H. pylori* strains leading to varying disease outcomes (1). So far only a single report describes the effect of *H. pylori* on the NKG2D axis (73). Heat-killed *H. pylori* induced *MICA* gene expression but not *MICB*, driven by *H. pylori*-LPS stimulating TLR4 in gastric epithelial cell lines activating also the cytotoxicity of peripheral blood lymphocytes. An important mechanistic difference of our study is the use of live *H. pylori*, which does *not* stimulate TLR4, in contrast to *H. pylori-*LPS alone (74). Moreover, heat-killed *H. pylori* cannot produce the pathogenicity factors VacA and CagA, which we identified as the major drivers of NKG2D-L modulation.

Soluble release of NKG2D-Ls is mediated by proteolytic shedding via metalloproteases (40, 52, 75), or by release on EVs (42). Using the broad-spectrum metalloprotease inhibitor batimastat, we found that the *H. pylori*-induced release of soluble NKG2D-Ls was specifically mediated by metalloproteases in MKN28 and AGS cells. ADAM17 is the best studied metalloprotease in the context of NKG2D-L shedding. This host protease is embedded in the cell membrane and gets activated when dissociated from α5β1 integrin (76). Curiously, the CagL protein located on the tip of the T4SS apparatus was shown to bind α5β1 integrin and thereby dissociate and activate ADAM17 (34). In this study, deletion of either *cagL* or *cagA*, significantly abrogated induction of MICA shedding. Silencing of *ADAM17* had no significant impact on shedding frequencies, nor did silencing of *MMP*9, an additional host protease that was induced in *H. pylori* infection in a *cagA*/*cagL*-dependent manner (77). Whether other proteases or a synergism of certain proteases is responsible for MICA/B cleavage remains to be determined (52, 53). Bacterial proteases might also take part in NKG2D-L release since they are key virulence factors and modulate host cells in various ways (78). The main *H. pylori* protease HtrA, however, could not account for the observed shedding since it is a serine-protease, not sensitive to batimastat inhibition (79).

The *MICA* and *MICB* genes are located in the highly polymorphic major histocompatibility complex (MHC)-class I locus and originate from a gene duplication event (80, 81). The two genes are highly homologous, but display numerous mutations and 531 *MICA* alleles and 244 *MICB* alleles are currently known (http://hla.alleles.org/alleles/classo.html, March 2023) (82). These genetic variations were shown to affect *MICA/B* expression and regulation (18, 51, 83–89). We detected lower baseline gene expression and greater inducibility of *MICB* compared to *MICA* in the cell lines AGS and MKN28 (**Supplementary Figure 4**). Regulatory differences in NKG2D-L expression might result from evolutionary selective pressure, in response to the evasion strategies of pathogens and tumors. In addition, these variations might allow for a diversified reaction of the system to variable stressors (6). Interestingly, specific *MICA/B* alleles are associated with certain diseases such as coeliac disease (90), ulcerative colitis (91), but also cancer, with gastric adenocarcinoma being associated with the *MICA* alleles A9 and *009/049 (80, 92). MICA A9 might show increased sensitivity to protease shedding due to a long transmembrane domain near the proteolytic cleavage site (52, 92, 93). In contrast, the frequent allele MICA*008 (A5.1), that is found in 20-55% of humans with varying frequencies in different populations (42, 82, 94–96), is less susceptible to proteolytic-shedding because of a truncated transmembrane domain (**Supplementary Figure 8**) (42). Thus, specific *MICA/B* alleles might differ in their susceptibility to *H. pylori*-induced shedding and this could influence the risk of developing *H. pylori*-induced pathologies, including gastric cancer (92).

In conclusion, our study shows that *H. pylori* actively manipulates the NKG2D system. This could help to protect the epithelial site of bacterial colonization from immune attack and thus shield *H. pylori* from eradication and contribute to its persistent colonization in the gastric mucosa. Furthermore, this mechanism might facilitate the development of stomach cancer by reducing the tumor surveillance represented by NK cells and CTLs (97). Our study provides a novel example of active immunoreceptor and/or -ligand modulation by the microbiota, similar to programmed cell death 1 ligand 1 (PD-L1) modulation by *H. pylori* (98), or proteolytic cleavage of Toll-like receptors (TLRs) by microbes (99). Our data provide new insights into the molecular principles governing microbe-induced proteolytic ectodomain shedding and enhance our knowledge regarding infection strategies employed by pathogens. Furthermore, these findings support a better characterization of tumor immune escape mechanisms, which could fuel the development of immunotherapy approaches.

## Data availability statement

The original contributions presented in the study are included in the article/**Supplementary Material**. Further inquiries can be directed to the corresponding author.

## Ethics statement

Ethical approval was not required for the studies on humans in accordance with the local legislation and institutional requirements because only commercially available established cell lines were used. Tissue use was approved by the institutional review board of the Medical University of Graz (EK-23-212ex10/11).

## Author contributions

MA: Data curation, Formal analysis, Investigation, Methodology, Visualization, Writing – original draft, Writing – review & editing. MW: Formal analysis, Investigation, Writing – review & editing. RH: Formal analysis, Investigation, Writing – review & editing. SE: Formal analysis, Methodology, Writing – review & editing. SW: Formal analysis, Investigation, Writing – review & editing. MM: Formal analysis, Investigation, Writing – review & editing. LS: Formal analysis, Investigation, Writing – review & editing. IK: Methodology, Writing – review & editing. SS: Methodology, Writing – review & editing. BJ: Methodology, Writing – review & editing. SK: Methodology, Supervision, Writing – review & editing. EZ: Methodology, Supervision, Writing – review & editing. GP: Methodology, Resources, Writing – review & editing. MV-G: Conceptualization, Methodology, Resources, Supervision, Writing – review & editing. HR: Methodology, Resources, Supervision, Writing – review & editing. GG: Conceptualization, Formal analysis, Funding acquisition, Investigation, Project administration, Supervision, Visualization, Writing – original draft, Writing – review & editing.
