# Supplementary material for: Immune evasion by proteolytic shedding of natural killer group 2, member D ligands in Helicobacter pylori infection
Source: Front Immunol. 2024 Jan 22;15:1282680. doi: 10.3389/fimmu.2024.1282680 (PMC10839011; doi:10.3389/fimmu.2024.1282680)
Supplement: Supplementary file 1 [file DataSheet_1.pdf]

Supplementary Material

1 Supplementary Results

1.1 Analysis of proteolytic shedding in *H. pylori* infection

We aimed to identify the proteases responsible for NKG2DL shedding. MMP9, MMP14, ADAM9, ADAM10 and ADAM17 have been reported to shed MICA/B in different cell types (1–8). Of these, MMP9, ADAM10 and ADAM17 were reported to be expressed in the stomach and might, therefore, be induced by *H. pylori* infection (9–14). We tested protease gene expressions in HpG and healthy control biopsies, as well as in MKN28 and AGS cells, with and without *H. pylori* infection (Supplementary Figure S10A). *MMP9, MMP14, ADAM9, ADAM10* and *ADAM17* were all expressed in stomach tissues and cell lines (Supplementary Figure S10A). In HpG tissues, *MMP9* expression was greatly induced, whereas *ADAM17* was unchanged and *ADAM9*, *ADAM10* and *MMP14* expressions were reduced compared to healthy controls (Supplementary Figure S10A). In cell lines, *MMP9* expression was induced 45-fold in MKN28 cells and about 175-fold in AGS cells upon *H. pylori* infection, whereas other proteases were only slightly induced, compared to non-infected cells (Supplementary Figure S10A). Notably, infection of AGS cells with *ΔcagA* and Δ*cagL* mutants abolished the *H. pylori* WT-triggered induction of *MMP9* expression, whereas deletion of *vacA* only reduced this induction (Supplementary Figure S10B). Thus, since we had found that soluble release of MICA was *cagA/L*-dependent (Figure 3), MMP9 seemed a likely candidate for NKG2DL shedding. Next, we silenced *MMP9* and also *ADAM17*, a protease reported to be activated by *H. pylori* (14). AGS cells were transfected with the respective small interfering RNA (siRNA) knock-down constructs or non-targeting (nt) controls and after 48 h cells were infected with *H. pylori* WT for another 24 h. Subsequently, protease gene expression was determined by qPCR and levels of sMICB in cell culture supernatants were determined by ELISA (Supplementary Figure S10C). The knock-down almost completely abolished *MMP9* mRNA expression and reduced *ADAM17* mRNA expression by about half (Supplementary Figure S10C). However, neither of the two knock-downs affected the release of sMICB in non-infected and *H. pylori*-infected cells (Supplementary Figure S10C), suggesting that individual activities of ADAM17 and MMP9 are not essential for MICB shedding in AGS cells. Optimization of ADAM17 knockdown-conditions may be required for a definitive conclusion on the role of ADAM17 in MICB shedding. Thus, the identity of the protease(s) triggering the release of soluble MICA/B in stomach epithelia remains to be elucidated.

2 Supplementary Materials and Methods

2.1 Isolation of EV-enriched preparations

To obtain EV-enriched preparations, EV-free FBS and HBS buffers were prepared by sequential ultra-centrifugation at 10.000 g for 30 min and at 100.000 g overnight (355618 tubes, Optima L-90K ultracentrifuge, Ti 70 rotor, Beckman coulter). Cells were cultured in T145 dishes for 48 h, then washed with PBS and re-cultured in fresh medium containing 1% of EV-free FBS. Cells were infected with *H. pylori* at MOI 50. As controls, cells were cultured without *H. pylori*. After 48 h of infection, cell culture supernatants were centrifuged twice at 200 g for 5 min and twice at 500 g for 10 min, to pellet cell debris. Then supernatants were centrifuged at 10,000 g for 30 min (UniCen HR, 6.50 TF Rotor), to pellet larger vesicles. Next, the supernatants were filtered with 0.22 µm filter systems (Corning),

transferred to ultracentrifugation tubes (326823, Beckman Coulter) and ultra-centrifugated at 100.000 g for 2 h (LE-80 Ultracentrifuge, SW28 rotor, Beckman Coulter). All centrifugation steps were performed at 4°C. The pellets were solubilized in EV-free HBS buffer (10 mmol/L HEPES pH 7.6, 150 mmol/L NaCl) and stored at -80°C. These preparations are referred to as EV-enriched preparations. The supernatants that remained after 100.000 g ultracentrifugation are referred to as EV-free supernatants.

2.2 Isolation of soluble proteins from EV-free supernatants

Soluble proteins were recovered from the EV-free supernatants by Trichloroacetic acid (TCA) precipitation. For this purpose, TCA (Sigma Aldrich) was added to the samples to a final concentration of 20%. After incubation at -20°C overnight, the samples were thawed and centrifuged at 12,000 g for 30 min at 4°C (UniCen HR, 6.50 TF Rotor). The pellets were washed once with PBS and twice with acetone. After air drying at room temperature the pellets were solubilized in 8 mol/L urea in PBS and stored at -80°C.

2.3 Visualization of EVs by electron microscopy

Visualization of EVs was achieved by electron microscopy. For this purpose, EV-enriched preparations were dropped on a parafilm and a grid was put on it for 1 min. Superfluous fluid was removed, and the sample was air-dried. The grid was placed on a drop of 2% uranylacetate 70% methanol and incubated for 20 min at RT. Several beakers were prepared: one with 15 ml methanol, one with 10 ml methanol, one with 5 ml methanol and one with fresh molecular-biology grade (MBG) water (Fresenius). The grid was put 8x in every beaker, then superfluous liquid was removed and the grid was dried for 5 min at RT. A drop of lead citrate (ready to use, Science Services) was placed on the grid and incubated for 1 min. Four beakers were filled with fresh MBG water and the grid was put 8x in every beaker. Superfluous liquid was removed and grids were dried and analyzed with a ZEISS EM 900 electron microscope.

2.4 Analysis of cell lysates, soluble proteins and EVs by western blot

Total proteins from cells were isolated by cell lysis with RIPA buffer (Merck Millipore) containing protease inhibitors 0.1 mmol/L Pefabloc, 1 mmol/L DTT, cOmplete Mini EDTA-free and PhosSTOP (Roche). Protein concentrations from cell lysates and soluble proteins were determined using the BioRad Protein Assay Dye Reagent (BioRad Laboratories). 20 µg of each protein sample were applied for western blot analysis. EVs were quantified by particle counts via a NanoSight device according to manufacturer’s specification. 5.319*10^8 particles of each EV sample were applied for western blot analysis. All samples were digested with PNGase F (New England Biolabs) for deglycosylation, according to manufacturer’s instructions. Subsequently, samples were mixed with loading buffer (Laemmli buffer, Biorad; diluted 1:9 with β-mercaptoethanol, Sigma-Aldrich), heated up to 95°C for 10 min, loaded onto 12,5% (v/v) (SDS)-polyacrylamide gels, subjected to electrophoresis and then blotted onto PVDF membranes (Immobilon-P, Merck Millipore). Blotting efficiency was determined by staining with Ponceau S solution (Sigma Aldrich). Non-specific binding was blocked for 1 h with 5% (w/v) non-fat dry milk (Bio-Rad Laboratories) in tris-buffered saline (TBS) plus 0.1% (v/v) Tween 20 (Merck Millipore). Subsequently, the membranes were incubated with the primary antibodies MICA biotinylated antibody (R&D Systems Cat# BAF1300, RRID: AB_355943, 1:2000) and MICB biotinylated antibody (R&D Systems Cat# BAF1599, RRID: AB_356059, 1:2000) overnight at 4°C, followed by incubation with streptavidin-HRP (R&D Systems

DY998, 1:5000) at RT for 1 h. For loading control, membranes were incubated with GAPDH antibody (Cell Signaling Technology #2118, 1:1000), overnight at 4°C, followed by incubation with rabbit IgG HRP linked F(ab′)2 (Merck, GENA9340-1ML, 1:5000) at RT for 1 h. Immunolabeling was detected using the ECL™ Select western blotting detection reagent (Merck, GERPN2235) and visualized with the ImageQuant™ LAS 500.

2.5 siRNA transfection

MMP9 and *ADAM17* were knocked down by siRNA transfection. For each protease, we tested a set of four different targeting siRNAs (ON-TARGET plus siRNAs, LQ-005970-00-0005 and LQ-003453-00-0002, Horizon Discovery) to identify the one with the best knockdown efficiency within our experimental setup (Supplementary Figure S2). Finally, siRNAs targeting *MMP9* (#4, 5'GAACCAAUCUCACCGACAG-3,) and *ADAM17* (#3, 5'-UAUGGGAACUCUUGGAUUA-3') were added to AGS cells in 6-well culture plates at 25 nmol/L, together with DharmaFECT 1 transfection reagent (T-2001-01, Horizon Discovery). A non-targeting siRNA (5'-UGGUUUACAUGUCGAUAA-3, D-001810-01-05, Horizon Discovery) was used as control. After 24 h, the cells were washed with medium and cultured for further 24 h. Subsequently, the cells were infected with *H. pylori* WT at MOI 50 for 24 h. As controls, cells were cultured without *H. pylori* for the same time.

2.6 Analysis of ADAM17 and ADAM10 in cell lysates by western blot

Protein isolation and western blotting were performed as described above. After blocking, the membranes were incubated with primary antibody against ADAM17 (Cell Signaling Technology Cat# 3976, RRID: AB_2242380, 1:2000) overnight at 4°C. Thereafter, membranes were washed and incubated with the Rabbit IgG HRP Linked F(ab′)2 (Merck, GENA9340-1ML, 1:5000) at RT for 1 h. Immunolabeling was detected using the ECL™ Select Western Blotting Detection Reagent (Merck, GERPN2235) and visualized with the ImageQuant™ LAS 500. Subsequently, membranes were treated with stripping buffer (Thermo Fisher, Restore™ Western blot Stripping Buffer) for 45 min at RT. Then membranes were washed, blocked and incubated with ADAM10 (Cell Signaling Technology Cat# 14194, RRID: AB_2798420, 1:500) overnight at 4°C. After that, membranes were washed and incubated with the Rabbit IgG HRP Linked F(ab′)2 (Merck, GENA9340-1ML, 1:5000) at RT for 1 h followed by immunolabelling and detection. Subsequently, membranes were again stripped, washed, blocked and then incubated with GAPDH (Cell Signaling Technology Cat# 2118, RRID: AB_561053, 1:1000) overnight at 4°C. Thereafter, membranes were washed and incubated with the Rabbit IgG HRP Linked F(ab′)2 (Merck, GENA9340-1ML, 1:5000) at RT for 1 h followed by immunolabelling and detection.

2.7 Target cell killing assay

To determine, whether supernatants of healthy and HP-infected epithelial cells affect NK cell cytotoxicity (target cell killing), we performed a cytotoxicity assay, according to Fernández-Messina et al. and Nociari et al. (15,16). Briefly, NK cell line NKL was incubated with filter-sterilized and filter-concentrated (5x) cell culture supernatants from non-infected AGS-MICA cells ('non-infected supernatant') and from H. pylori-infected AGS-MICA cells ('H. pylori-infected supernatant') for 24 h. To determine the effect of these treatments on NK cell cytotoxicity, NKL cells were then were washed and co-cultivated with K562 cells at an effector:target ratio of 3:1 for 6 h, followed by a measurement of cell viability using resazurin. The percentage of specific lysis of target cells was

calculated as 100×{(AF of targets alone)–[(AF of mix)–(AF of effectors alone)]}/AF of targets alone, where AF=absolute fluorescence units.

3 Supplementary References

1. Boutet P, Agüera-González S, Atkinson S, Pennington CJ, Edwards DR, Murphy G, Reyburn HT, Valés-Gómez M. Cutting Edge: The Metalloproteinase ADAM17/TNF-α-Converting Enzyme Regulates Proteolytic Shedding of the MHC Class I-Related Chain B Protein. *J Immunol* (2009) 182:49–53. doi: 10.4049/jimmunol.182.1.49

2. Yamanegi K, Yamane J, Kobayashi K, Ohyama H, Nakasho K, Yamada N, Hata M, Fukunaga S, Futani H, Okamura H, et al. Downregulation of matrix metalloproteinase-9 mRNA by valproic acid plays a role in inhibiting the shedding of MHC class I-related molecules A and B on the surface of human osteosarcoma cells. *Oncol Rep* (2012) 28:1585–1590. doi: 10.3892/or.2012.1981

3. Shiraishi K, Mimura K, Kua LF, Koh V, Siang LK, Nakajima S, Fujii H, Shabbir A, Yong WP, So J, et al. Inhibition of MMP activity can restore NKG2D ligand expression in gastric cancer, leading to improved NK cell susceptibility. *J Gastroenterol* (2016) 51:1101–1111. doi: 10.1007/s00535-016-1197-x

4. Liu G, Atteridge CL, Wang X, Lundgren AD, Wu JD. Cutting Edge: The Membrane Type Matrix Metalloproteinase MMP14 Mediates Constitutive Shedding of MHC Class I Chain-Related Molecule A Independent of A Disintegrin and Metalloproteinases. *J Immunol* (2010) 184:3346–3350. doi: 10.4049/jimmunol.0903789

5. Kohga K, Takehara T, Tatsumi T, Ishida H, Miyagi T, Hosui A, Hayashi N. Sorafenib inhibits the shedding of major histocompatibility complex class i-related chain a on hepatocellular carcinoma cells by down-regulating a disintegrin and metalloproteinase 9. *Hepatology* (2010) 51:1264–1273. doi: 10.1002/hep.23456

6. Waldhauer I, Goehlsdorf D, Gieseke F, Weinschenk T, Wittenbrink M, Ludwig A, Stevanovic S, Rammensee HG, Steinle A. Tumor-associated MICA is shed by ADAM proteases. *Cancer Res* (2008) 68:6368–6376. doi: 10.1158/0008-5472.CAN-07-6768

7. Chitadze G, Lettau M, Bhat J, Wesch D, Steinle A, Fürst D, Mytilineos J, Kalthoff H, Janssen O, Oberg HH, et al. Shedding of endogenous MHC class I-related chain molecules A and B from different human tumor entities: Heterogeneous involvement of the "a disintegrin and metalloproteases" 10 and 17. *Int J Cancer* (2013) 133:1557–1566. doi: 10.1002/ijc.28174

8. Kohga K, Takehara T, Tatsumi T, Miyagi T, Ishida H, Ohkawa K, Kanto T, Hiramatsu N, Hayashi N. Anticancer chemotherapy inhibits MHC class I-related chain A ectodomain shedding by downregulating ADAM10 expression in hepatocellular carcinoma. *Cancer Res* (2009) 69:8050–8057. doi: 10.1158/0008-5472.CAN-09-0789

9. Mori N, Sato H, Hayashibara T, Senba M, Geleziunas R, Wada A, Hirayama T, Yamamoto N. Helicobacter pylori induces matrix metalloproteinase-9 through activation of nuclear factor κB. *Gastroenterology* (2003) 124:983–992. doi: 10.1053/gast.2003.50152

10. Oliveira MJ, Costa AC, Costa AM, Henriques L, Suriano G, Atherton JC, Machado JC, Carneiro F, Seruca R, Mareel M, et al. Helicobacter pylori induces gastric epithelial cell invasion in a c-Met and type IV secretion system-dependent manner. *J Biol Chem* (2006) 281:34888–34896. doi: 10.1074/jbc.M607067200

11. Yoshimura T, Tomita T, Dixon MF, Axon ATR, Robinson PA, Crabtree JE. ADAMs (A disintegrin and metalloproteinase) messenger RNA expression in Helicobacter pylori-infected, normal, and neoplastic gastric mucosa. *J Infect Dis* (2002) 185:332–340. doi: 10.1086/338191

12. Hoy B, Löwer M, Weydig C, Carra G, Tegtmeyer N, Geppert T, Schröder P, Sewald N, Backert S, Schneider G, et al. Helicobacter pylori HtrA is a new secreted virulence factor that cleaves E-cadherin to disrupt intercellular adhesion. *EMBO Rep* (2010) 11:798–804. doi: 10.1038/embor.2010.114

13. McClurg UL, Danjo K, King HO, Scott GB, Robinson PA, Crabtree JE. Epithelial cell ADAM17 activation by Helicobacter pylori: Role of ADAM17 C-terminus and Threonine-735 phosphorylation. *Microbes Infect* (2015) 17:205–214. doi: 10.1016/j.micinf.2014.11.011

14. Saha A, Backert S, Hammond CE, Gooz M, Smolka AJ. Helicobacter pylori CagL Activates ADAM17 to Induce Repression of the Gastric H, K-ATPase α Subunit. *Gastroenterology* (2010) 139:239–248. doi: 10.1053/j.gastro.2010.03.036

15. Fernández-Messina L, Ashiru O, Agüera-González S, Reyburn HT, Valés-Gómez M. The human NKG2D ligand ULBP2 can be expressed at the cell surface with or without a GPI anchor and both forms can activate NK cells. *J Cell Sci* (2011) 124:321-7. doi: 10.1242/jcs.076042.

16. Nociari MM, Shalev A, Benias P, Russo C. A novel one-step, highly sensitive fluorometric assay to evaluate cell-mediated cytotoxicity. *J Immunol Methods* (1998) 213(2):157-67. doi: 10.1016/s0022-1759(98)00028-3.

4 Supplementary Figures

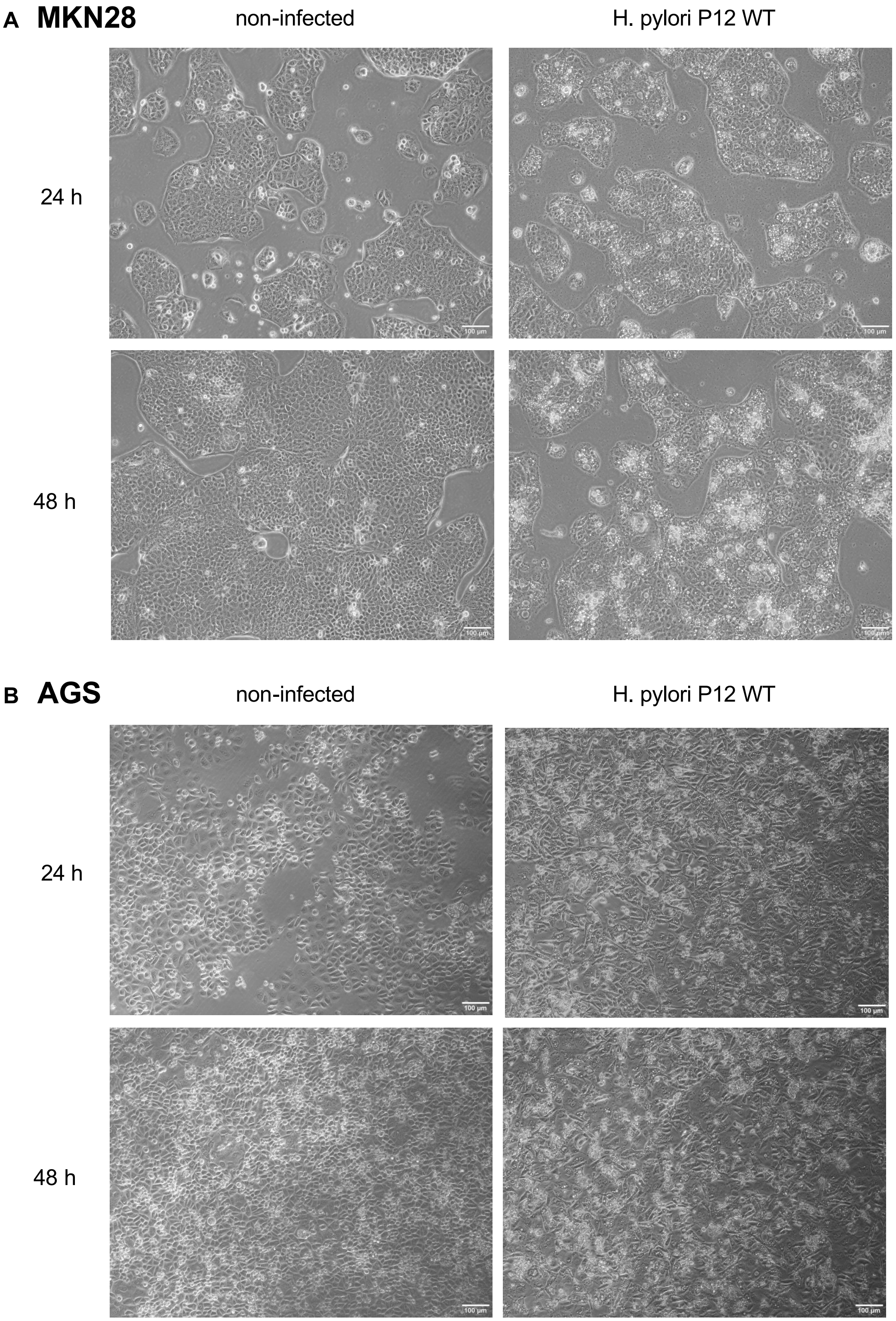

Supplementary Figure S1. Light microscopy of stomach epithelial cell lines MKN28 (a) and AGS (b) after infection with *H. pylori* P12 WT for 24 and 48 h.

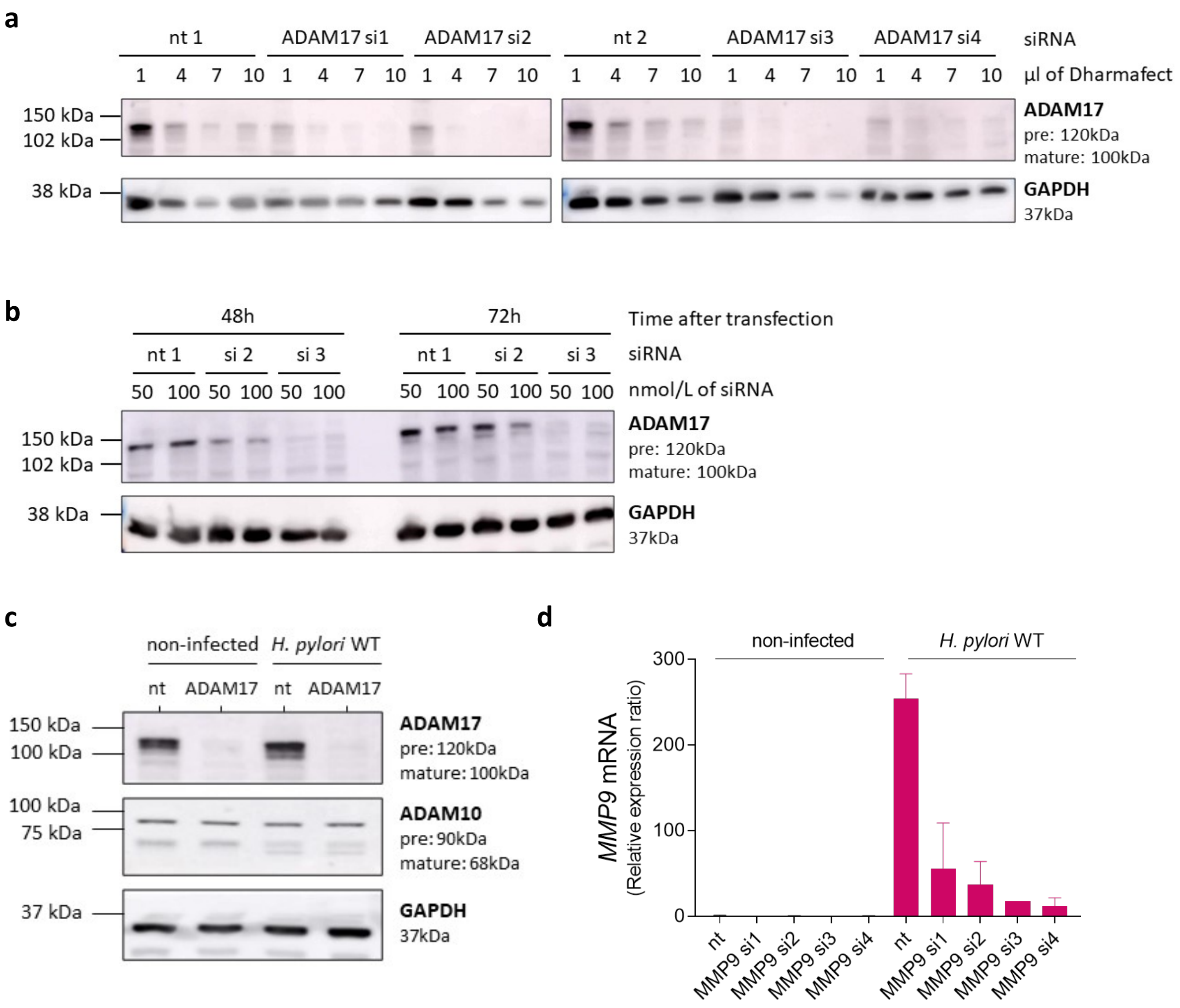

Supplementary Figure S2. Testing different conditions for the knockdown of *ADAM17* and *MMP9* in AGS cells. For the knockdown of *ADAM17* in AGS cells, knockdown efficacy was determined by Western Blot. (a) We tested two non-targeting (nt) siRNAs and four *ADAM17*-targeting siRNAs in combination with different volumes of Dharmafect (1, 4, 7 and 10µl), at different siRNA concentrations (50 nmol/L and 100 nmol/L) and (b) at different timepoints after transfection (48 h and 72 h) and (c) with versus without *H. pylori* WT infection. (d) For the knockdown of *MMP9* in AGS cells, knockdown efficacy was determined by qPCR, since MMP9 is a secreted protein. We tested one non-targeting (nt) and four *MMP9*-targeting siRNAs, with and without *H. pylori* WT infection.

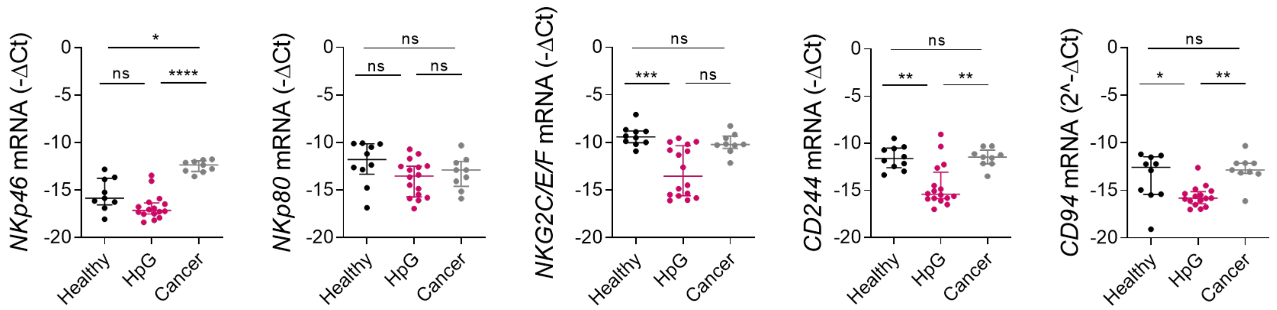

Supplementary Figure S3. Gene expression analysis of NK cell marker genes in stomach biopsies. Quantifying NK cells in tissues is challenging, first because NK cells share most marker genes and proteins with T cells, and second because NK cells are a versatile cell population and not all NK cells express the same markers at similar levels at all times. Therefore, to comparatively assess NK cell frequencies in gastric biopsies, we selected NK cell marker genes that are expected to be expressed in most NK cell subtypes and are only rarely found in T cells. Gene expression analysis of *NKp46, NKp80, CD94, CD244* and *NKG2C/E/*F was performed via qPCR, n=9-16 per group, the data do not follow a normal distribution, median ± interquartile range, Kruskal-Wallis test and Dunn's test. * P <0.05; ** P <0.01; *** P <0.001; **** P <0.0001.

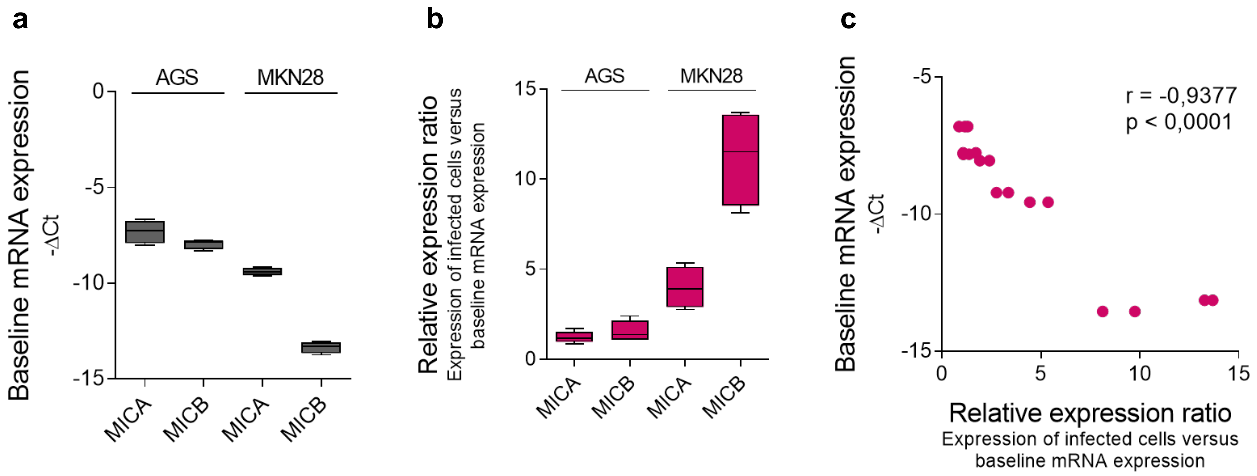

Supplementary Figure S4. Inducibility of *MICA* and *MICB* gene expression correlates with the level of baseline gene expression in AGS and MKN28 cells. (a) *MICA* and *MICB* mRNA expression levels of the cell lines MKN28 and AGS prior to any treatment (=baseline) were determined by qPCR analysis. The results are presented as -ΔCt, Box and Whiskers Min to Max. (b) MKN28 and AGS cells were infected with *H. pylori* WT for 48 h, *MICA* and *MICB* mRNA levels were determined by qPCR analysis. The results are presented as the relative expression ratio of infected cells versus baseline mRNA expression, Box and Whiskers Min to Max. (c) Correlation of the baseline gene expression and the relative expression ratio after infection for 48 h, Pearson correlation.

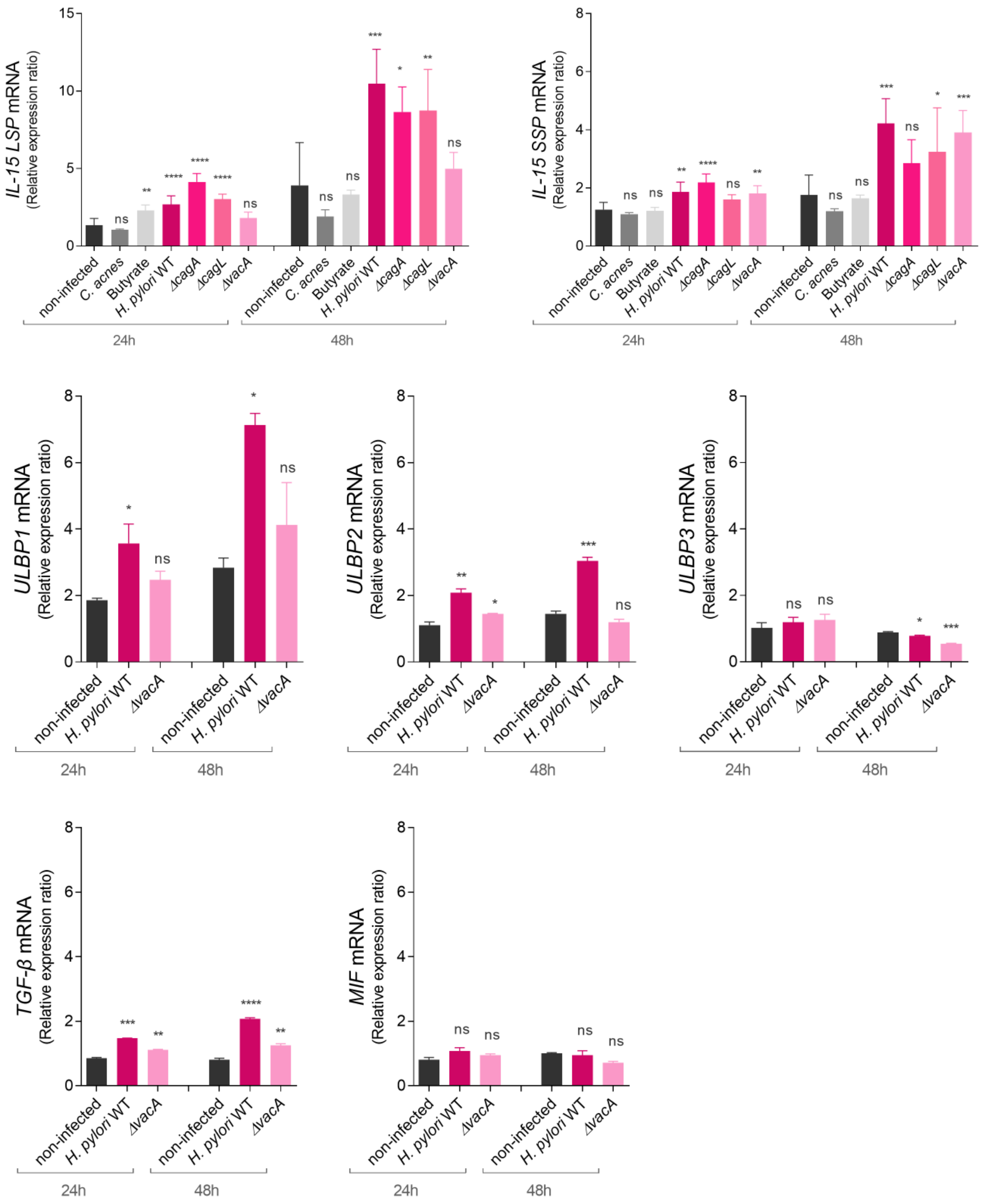

Supplementary Figure S5. Gene expression analysis of activators (*IL-15*, *ULBP1-3*) and inhibitors (*TGF-β, MIF*) of NKG2D in *H. pylori* infection in MKN28 cells. MKN28 cells were challenged with *C. acnes*, butyrate, *H. pylori* WT and isogenic mutants *ΔcagA*, *ΔcagL* and *ΔvacA*, for 24 and 48 h. Gene expression was determined by qPCR analysis. Mean +/- SD, one-way ANOVA and Dunnett's test, asterisks indicate statistically significant differences of treated cells compared to non-infected cells at the corresponding timepoint (ns = not significant, * P <0.05; ** P <0.01; *** P <0.001; **** P <0.0001).

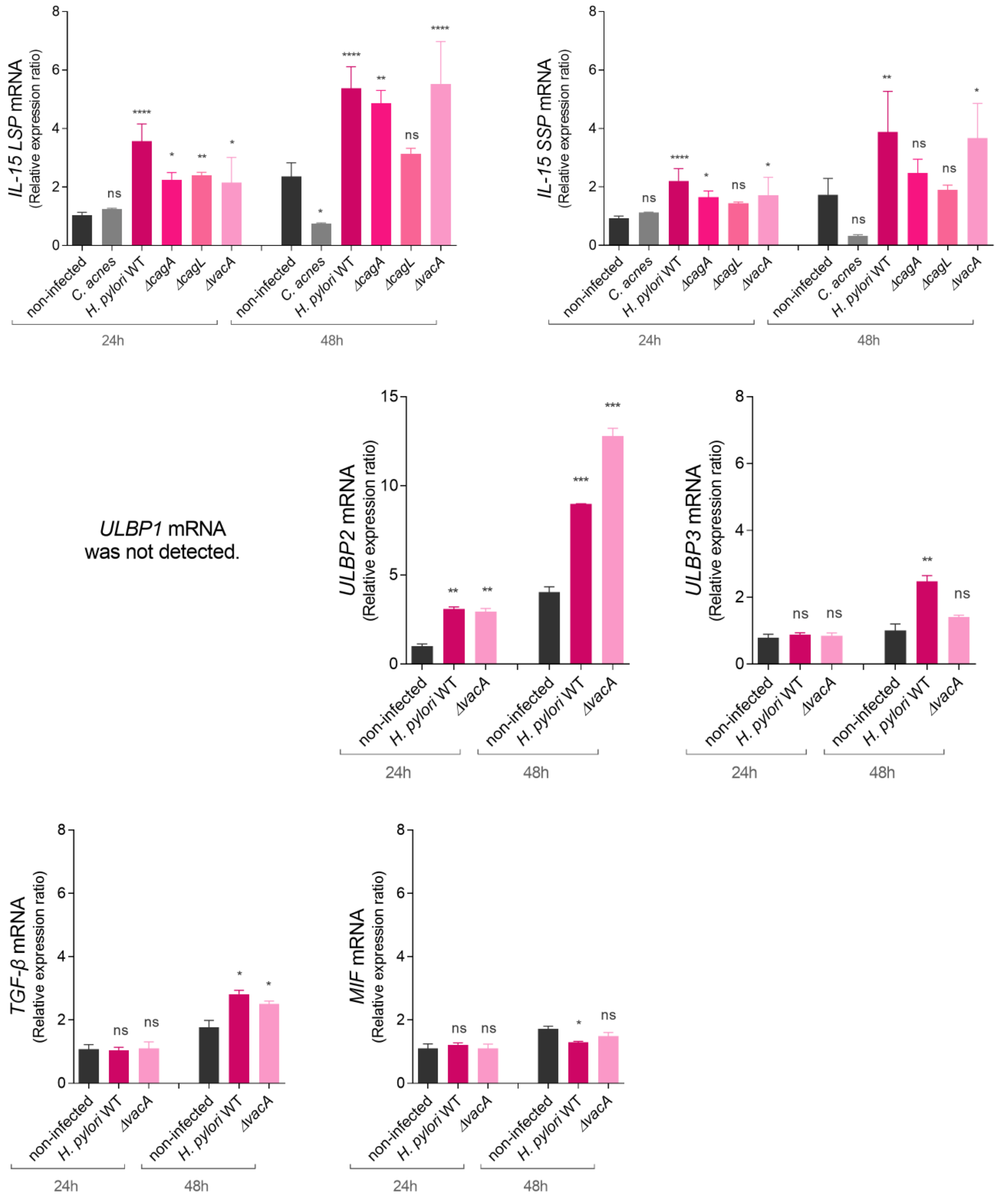

Supplementary Figure S6. Gene expression analysis of activators (*IL-15*, *ULBP1-3*) and inhibitors (*TGF-β, MIF*) of NKG2D in *H. pylori* infection in AGS cells. AGS cells were challenged with *C. acnes*, *H. pylori* WT and isogenic mutants *ΔcagA*, *ΔcagL* and *ΔvacA*, for 24 and 48 h. Gene expression was determined by qPCR analysis. Mean +/- SD, one-way ANOVA and Dunnett's test, asterisks indicate statistically significant differences of treated cells compared to non-infected cells at the corresponding timepoint (ns = not significant, * P <0.05; ** P <0.01; *** P <0.001; **** P <0.0001).

a

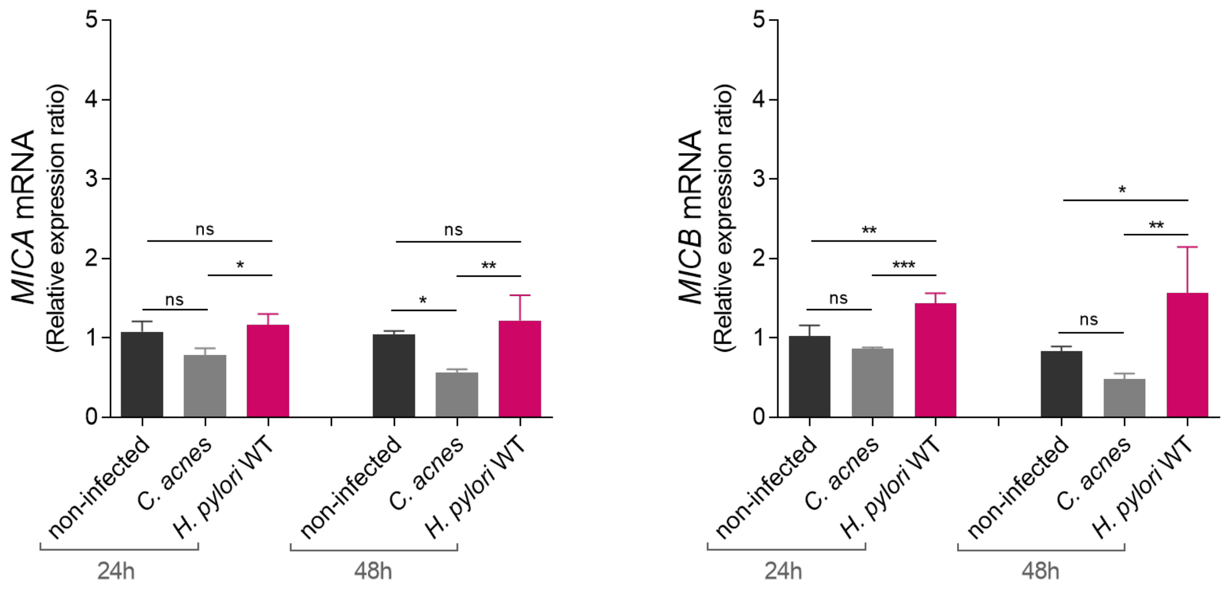

b

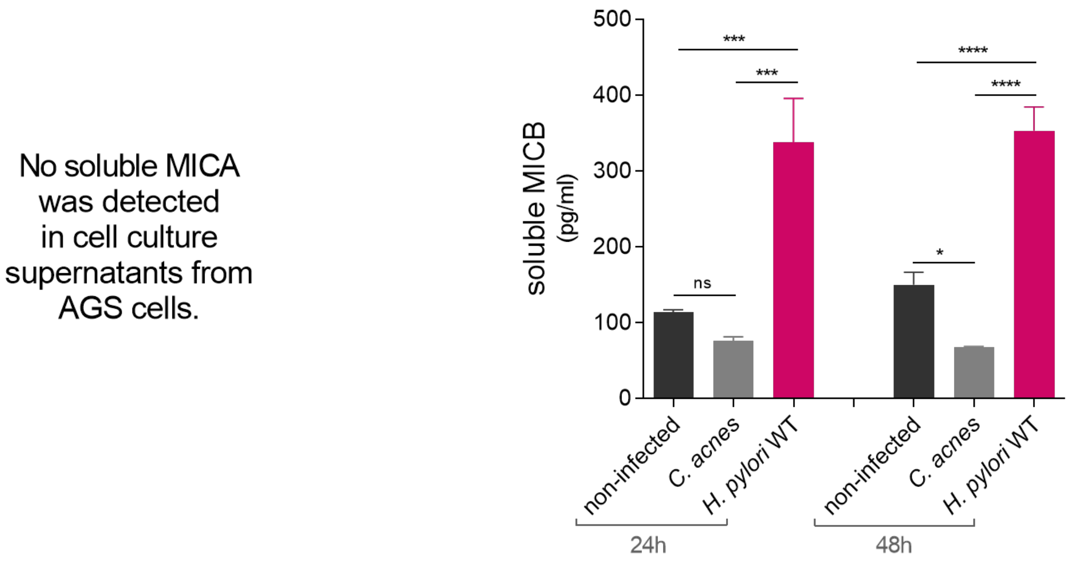

Supplementary Figure S7. Analysis of MICA and MICB expression in AGS cells. AGS cells were challenged with *C. acnes* and *H. pylori* WT for 24 and 48 h. (a) *MICA* and *MICB* mRNA levels were determined by qPCR. (b) Soluble MICA and MICB levels in cell culture supernatants were determined by ELISA. Mean +/- SD, one-way ANOVA and Tukey's test (ns = not significant, * P <0.05; ** P <0.01; *** P <0.001; **** P <0.0001).

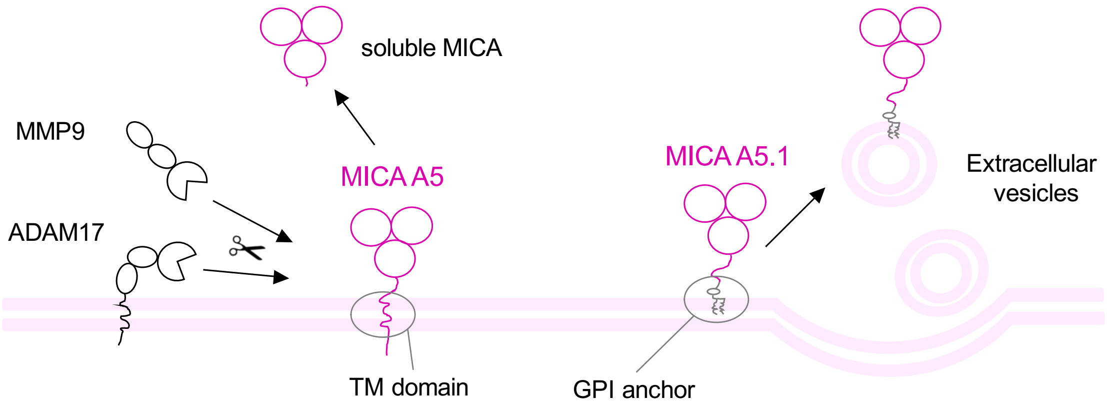

Supplementary Figure S8. Scheme of the differential mechanisms of NKG2DL release. MICA alleles with a full-length transmembrane (TM) domain, such as MICA A5, are released as soluble proteins via metalloprotease-mediated shedding. Truncated allele MICA A5.1 is attached to the membrane with a glycosylphosphatidylinositol (GPI) anchor. This allelic version cannot be cleaved by proteases, but is instead released in extracellular vesicles (EVs).

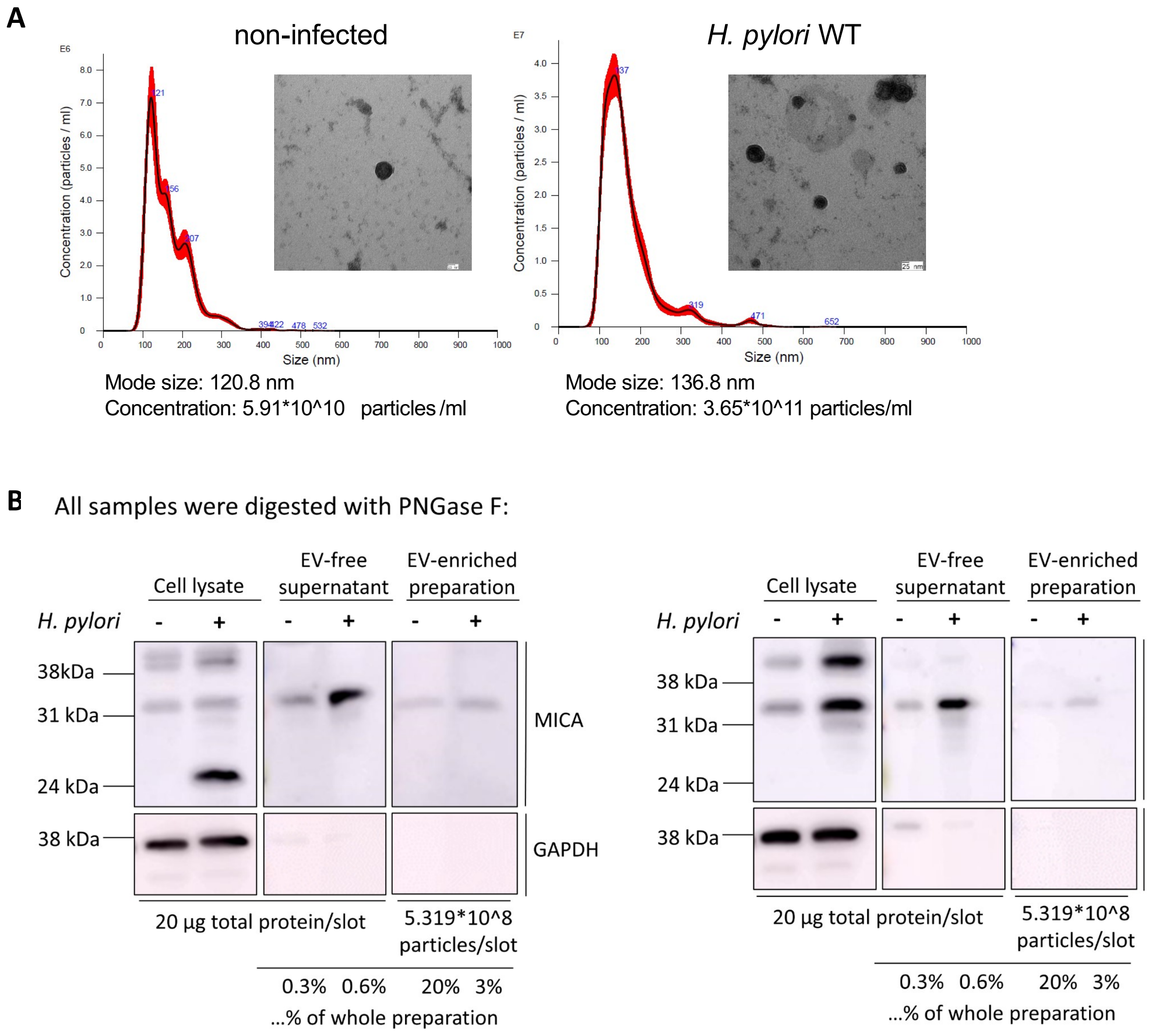

Supplementary Figure S9. Comparison of cell-associated, EV-associated and soluble MICA and MICB in supernatants of *H. pylori* infected stomach epithelial cells. (A-B) AGS cells were cultured with or without *H. pylori* WT for 48 h, then cell culture supernatants were subjected to a sequential centrifugation protocol to obtain EV-enriched preparations (= pellet after final centrifugation step at 100.000 g) and EV-free supernatants (= supernatant after final centrifugation step at 100.000 g). (A) EV-preparations were analyzed by Nanosight and Electron Microscopy. (B) Protein preparations from whole cell lysates, EV-enriched preparations and EV-free supernatants were digested with PNGase F for deglycosylation and then analyzed by Western Blot.

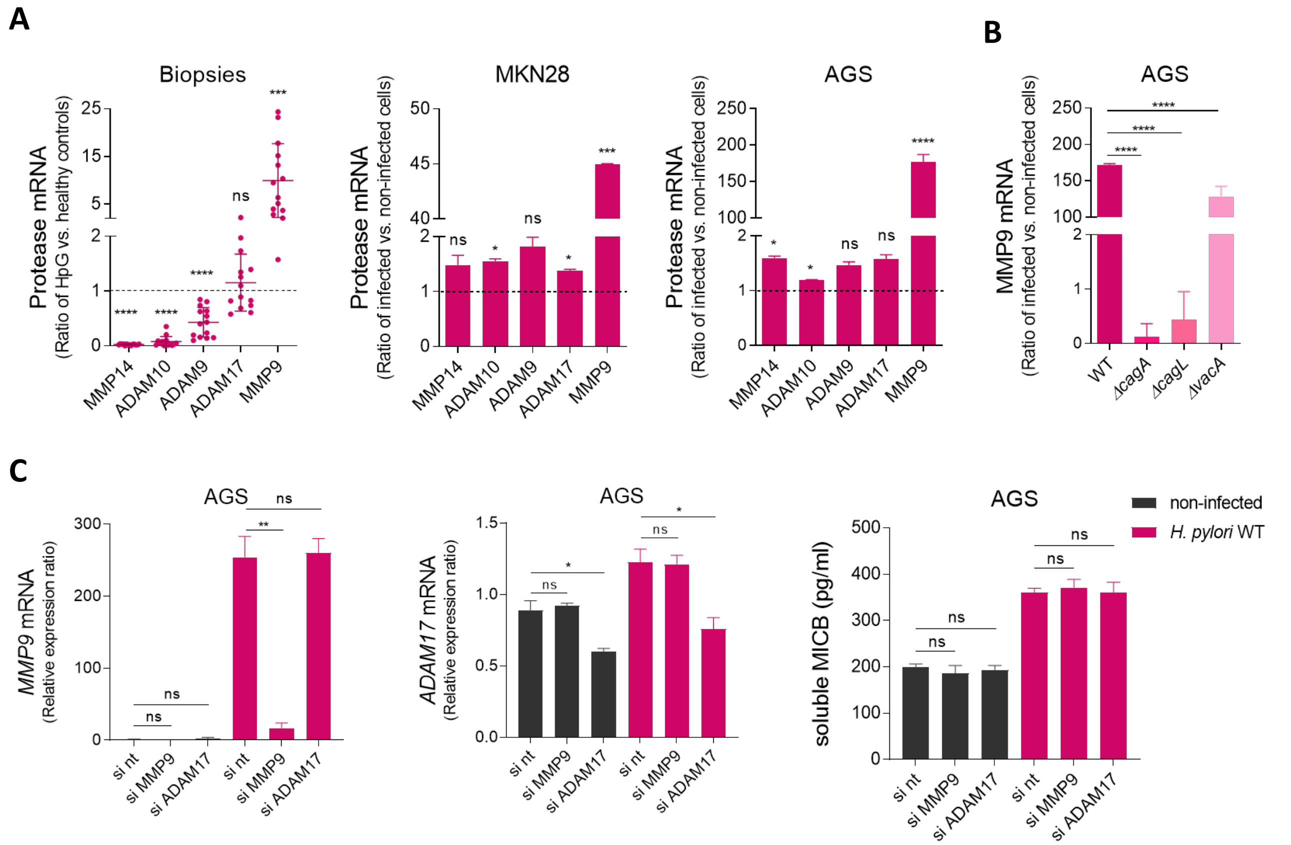

Supplementary Figure S10. Analysis of proteolytic shedding in *H. pylori* infection. (A) mRNA expression of *ADAM9, ADAM10, ADAM17, MMP9* and *MMP14* was determined in stomach biopsies of healthy controls and HpG cases (n=6-14 per group) and in MKN28 and AGS cells, non-infected and after *H. pylori* infection for 24 h. Cell culture experiments were performed three times. For biopsies, the results are shown as the relative expression ratio of HpG cases vs. the mean of all healthy control cases. For cell culture experiments, the results are shown as the relative expression ratio of infected cells vs. non-infected cells. Mean ± SD, one sample t-test vs. a theoretical mean of 1 (ns = not significant; * P <0.05; *** P <0.001; **** P <0.0001). (B) AGS cells were infected with *H. pylori* WT and isogenic mutants *ΔcagA, ΔcagL* and *ΔvacA* for 24 h. *MMP9* gene expression was determined by qPCR analysis. qPCR data are shown as the relative expression ratio of treated cells vs. cells harvested at time point 0 h. Experiments were performed three times, one-way ANOVA and Dunnett's test (**** P <0.0001). (C) AGS cells were transfected with a non-targeting (nt) siRNA and siRNAs targeting *MMP9* and *ADAM17*. After 48 h, cells were infected with *H. pylori* for 24 h. Subsequently, protease gene expression was determined by qPCR and sMICB in cell culture supernatants was determined by ELISA. qPCR data are shown as the relative expression ratio of treated cells vs. cells harvested at time point 0 h. Mean ± SD, one-way ANOVA and Dunnett's test (ns = not significant, * P <0.05; ** P <0.01).

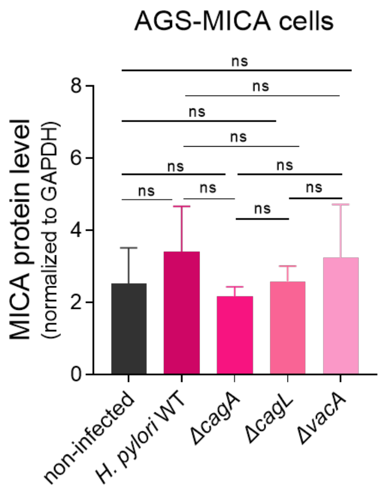

Supplementary Figure S11. *H. pylori* virulence factor-dependent modulation of MICA protein. AGS-MICA cells were challenged with *H. pylori* WT and isogenic mutants *ΔcagA*, *ΔcagL* and *ΔvacA* for 24 h. MICA protein in cell lysates was determined by Western Blot. Experiments were performed three times. Mean ± SD, one-way ANOVA and Tukey's test (ns = not significant, * P <0.05; ** P <0.01; *** P <0.001; **** P <0.0001).

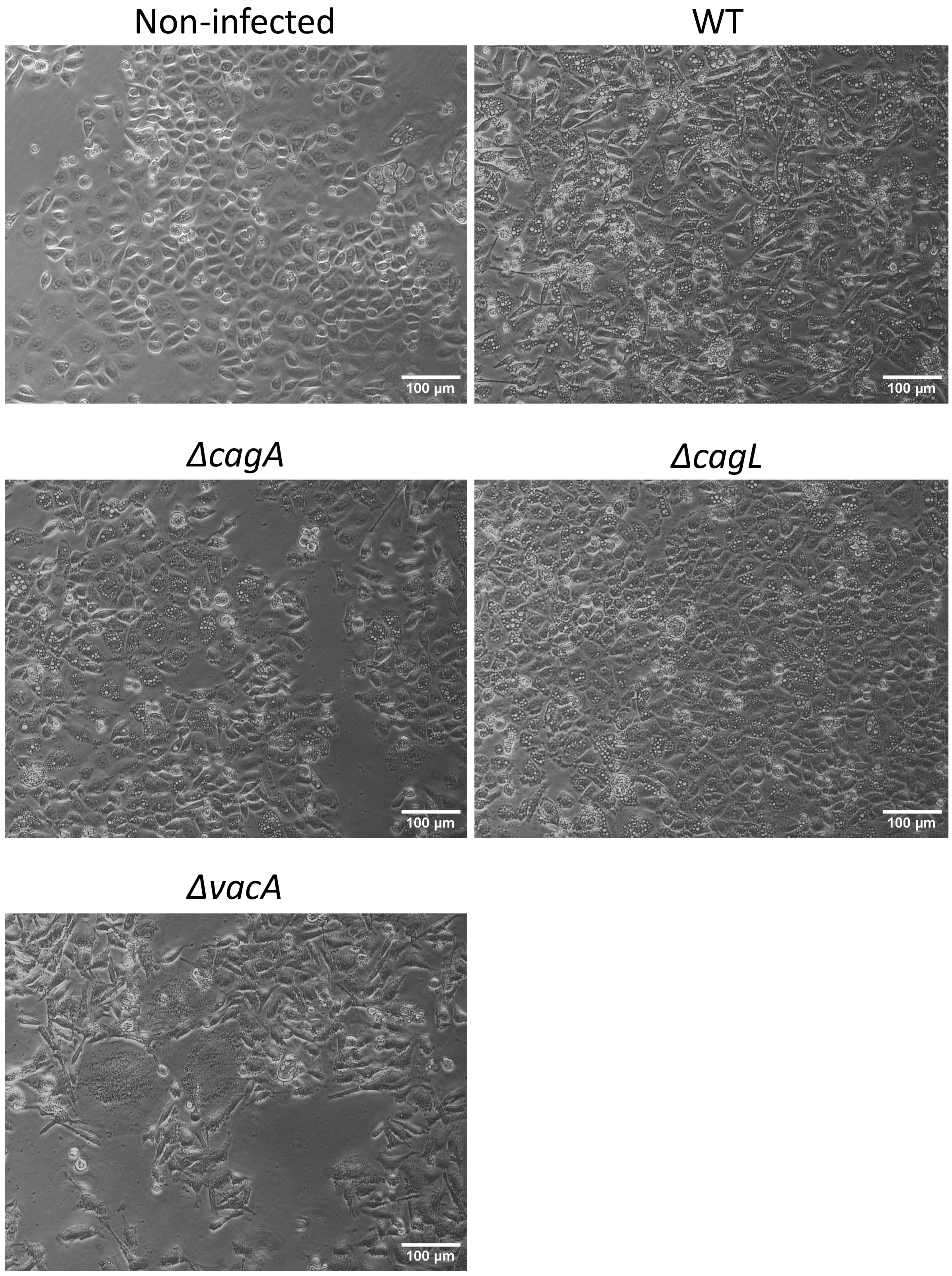

Supplementary Figure S12. Light microscopy of stomach epithelial cell line AGS after infection with *H. pylori* P12 WT and the isogenic mutants *ΔcagA*, *ΔcagL* and *ΔvacA* for 24 h.

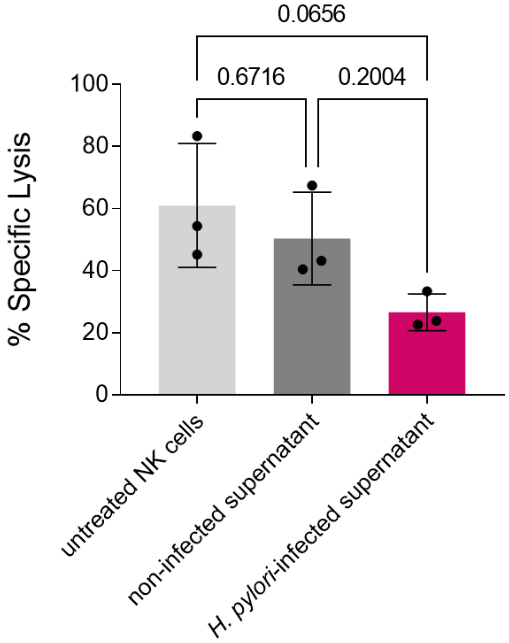

Supplementary Figure S13. Analysis of the effect of cell culture supernatants from non-infected and *H. pylori*-infected gastric epithelial cells on the cytotoxicity of NK cells. NKL cells were incubated with cell culture supernatants from non-infected AGS-MICA cells ('non-infected supernatant') and from *H. pylori*-infected AGS-MICA cells ('H. pylori-infected supernatant') for 24 h. NKL cells were co-cultivated with K562 target cells at an effector:target ratio of 3:1 for 6 h, followed by a measurement of cell viability using resazurin. The percentage of specific lysis of target cells was calculated. The experiment was performed three times (mean, SD and all points, one-way ANOVA and Tukey's test).
